# Deep-Blue Triplet–Triplet Annihilation Organic Light-Emitting Diode (CIE_y_ ≈ 0.05) Using Tetraphenylimidazole and Benzonitrile Functionalized Anthracene/Chrysene Emitters

**DOI:** 10.3390/molecules27248923

**Published:** 2022-12-15

**Authors:** Ruttapol Malatong, Wijitra Waengdongbung, Phattananawee Nalaoh, Nuttapong Chantanop, Pongsakorn Chasing, Chokchai Kaiyasuan, Suangsiri Arunlimsawat, Taweesak Sudyoadsuk, Vinich Promarak

**Affiliations:** Department of Materials Science and Engineering, School of Molecular Science and Engineering, Vidyasirimedhi Institute of Science and Technology, Wangchan, Rayong 21210, Thailand

**Keywords:** anthracene, chrysene, deep-blue emitters, triplet–triplet annihilation, organic light-emitting diode

## Abstract

Herein, new deep-blue triplet-triplet annihilation (TTA) molecules, namely 4-(10-(4-(1,4,5-triphenyl-1H-imidazol-2-yl)phenyl)anthracen-9-yl)benzonitrile (TPIAnCN) and 4-(12-(4-(1,4,5-triphenyl-1H-imidazol-2-yl)phenyl)chrysen-6-yl)benzonitrile (TPIChCN), are designed, synthesized, and investigated as emitters for organic light-emitting diodes (OLED). TPIAnCN and TPIChCN are composed of polyaromatic hydrocarbons of anthracene (An) and chrysene (Ch) as the cores functionalized with tetraphenylimidazole (TPI) and benzonitrile (CN) moieties, respectively. The experimental and theoretical results verify their excellent thermal properties, photophysical properties, as well as electrochemical properties. Particularly, their emissions are in the deep blue region, with TTA emissions being observed in their thin films. By utilization of these molecules as emitters, deep blue TTA OLEDs with CIE coordinates of (0.15, 0.05), high external quantum efficiency of 6.84%, and high exciton utilization efficiency (η_s_) of 48% were fabricated. This result manifests the potential use of chrysene as an alternate building block to formulate new TTA molecules for accomplishing high-performance TTA OLEDs.

## 1. Introduction

Organic light-emitting diodes (OLEDs) have been widely studied since the publication of the pioneering report in 1978 by Tang et al. [1] owing to their attractive characteristics (transparent lighting panels, high brightness, color tuneability, as well as flexibility) and potential applications in the new generation of display and solid-state lighting technologies [2,3,4,5,6]. In particular, blue light-emitting OLEDs are indispensable as one of the three primary colors (blue, green, and red) for full-color display. However, in terms of device performance, blue OLEDs still lag behind both red and green OLEDs to date. This is because of the extrinsically wide energy band gap of blue emitters, complicating the molecular design and the discovery of the well-matched hole transporting layer or electron transporting layer [7,8,9]. Hence, the development of blue emitters for blue OLEDs is very fascinating and challenging to study for the improvement of device performance. Due to the limit of efficiency for blue fluorescent OLEDs with the theoretical maximum external quantum efficiency (EQE_max_) of 5%, harvesting only singlet excitons [10,11,12,13,14], there are currently several blue OLED mechanisms for enhancing the device performance to harvest the triplet excitons, such as phosphorescence, thermally activated delayed fluorescence (TADF) [15,16,17,18], hybridized local charge transfer (HLCT) [19,20], and triplet-triplet annihilation (TTA) [21,22]. These mechanisms have achieved EQEs of higher than 5%. However, the metal-containing blue phosphorescent materials barely give deep-blue emissions attributable to their radiative metal-ligand charge transfer (MLCT) nature. Moreover, these emitters are not environmentally friendly and cost-effective when scaled up for applications [23,24], whereas TADF materials can accomplish the internal quantum efficiency (IQE) of 100% through reverse intersystem crossing from a triplet excited state to a singlet excited state. However, the strong donor and acceptor features in these emitters promote a potent charge transfer (CT) effect, resulting in sky-blue emission. Therefore, purely deep blue emitters remain an urgent concern and crucial to obtain fluorescent emitters that can realize high efficiency [25,26,27]. Moreover, TADF devices rapidly decline at high luminance, and device fabrication is complicated [25,26,28].

**Scheme 1:**
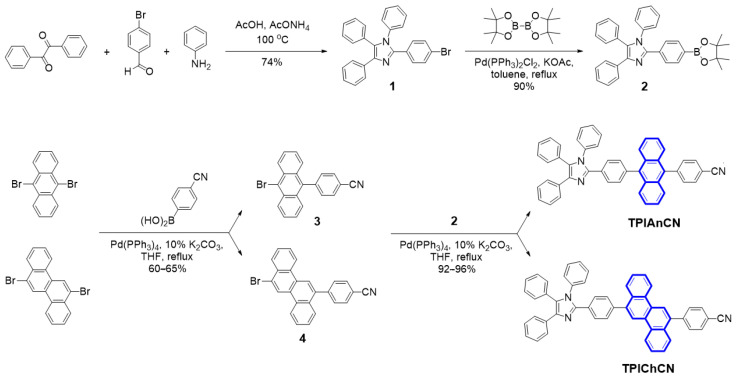
Synthetic routes to TPIAnCN and TPIChCN (highlighted in blue are the cores).

Recently, triplet-triplet annihilation (TTA) is one of the most encouraging systems, harvesting the triplet excitons which can achieve the IQE of 62.5% through the fusion of the two triplets (T_1_) individual molecules. The excited state energy of 2T_1_ should be higher than S_1_, consequently producing the amount of singlet excited excitons for light emission [29]. Indeed, the advantages of deep blue TTA based-devices exhibited potential in low roll-off efficiency, long operational lifetime, and high color purity of EL emission [30,31,32,33]. However, the EQEs of blue devices are yet to be applied at a practical level. Thus, the development of highly efficient deep blue OLEDs is still pinching and needs more investigation. Until now, most efficient deep blue TTA materials were intensively considered and designed to achieve high efficiency, especially polyaromatic hydrocarbon-based deep blue TTA emitters, such as benzonitrile substituted anthracene derivatives [34,35,36,37,38]. In these molecules, anthracene with a large flat conjugated structure flavor bimolecular interactions and can stabilize the triplet pair, whereas the benzonitrile unit can enhance the molecular interactions facilitating the TTA process [39]. In addition, further modification on another side of benzonitrile substituted anthracene with various moieties provides tuning of the photophysical properties as well as device performances of the materials, such as imidazole, fluorene, arylamine, and polyaromatics. Imidazole is a five-membered heterocyclic aromatic ring. Owing to its electron-deficient nature, its derivatives exhibit intrinsic ambipolar properties [40,41,42]. 2-[4-(9,10-Di-naphthalen-2-yl-anthracen-2-yl)-phenyl]-1-phenyl-1H-benzoimidazole (ZADN), and 2,2′,2″-(1,3,5-benzinetriyl)-tri(1-phenyl-1H-benzimidazole (TPBi) as examples are well-known electron transporting materials used in OLEDs [43,44,45,46]. In addition, imidazole derivatives with bipolar electronic properties could balance charge transport in OLEDs, whereas rigid planar π-conjugation could reduce the non-radiative transitions of molecules, giving a high photoluminescence quantum yield (Φ_PL_) in solid state as well as good thermal and morphological stabilities to ensure the device stability [47]. In this work, we, therefore, designed and synthesized the highly efficient deep blue emitters based on different polyaromatic derivatives, anthracene (An) and chrysene (Ch) functionalized with tetraphenylimidazole (TPI) and benzonitrile (CN), namely TPIAnCN and TPIChCN, respectively (Scheme 1). The TPI moiety with a near-ultraviolet emission property due to its limited conjugation will provide tuning of the photophysical property. The benzonitrile unit will offer an enhanced strong molecular interaction to facilitate the TTA mechanism, while either anthracene or chrysene will serve as TTA building blocks as well as afford to tune deep blue light-emitting [48,49,50,51]. Besides, stable and efficient deep blue OLEDs with International Commission on Illumination (CIE) coordinates of (0.15, 0.06) that meet the requirement for high-definition television (HDTV) displays are still rare [9,19,52]. It is anticipated that the use of chrysene as a core would provide a new deep blue emission molecule [53]. Indeed, both TPIAnCN and TPIChCN give deep blue emission color with high solid-state fluorescence efficiency and a TTA property, and so, their OLEDs attain admirable EL performances (EQE_max_ = 5.31–6.84%) with high exciton utilization efficiency (η_s_) of 36–48% and CIE coordinates of (0.15, 0.05).

## 2. Results and Discussion

The synthesis of TPIAnCN and TPIChCN is outlined in Scheme 1. A one-pot reaction of benzil, 4-bromobenzaldehyde, and aniline followed by borylation of 1 with bis(pinacolato)diboron gave pinacol ester 2 in good yield over two steps. Two benzonitrile intermediates of 4-(10-bromoanthracen-9-yl)benzonitrile (3) and 4-(12-bromochrysen-6-yl)benzonitrile (4) were then prepared via a mono-palladium-catalyzed Suzuki cross-coupling reaction of 4-cyanophenyboronic acid with either 9,10-dibromoanthracene or 6,12-dibromochrysene. In the final step, the reactions of pinacol ester 2 with either bromo benzonitriles 3 or 4 using Pd(PPh_3_)_4_/K_2_CO_3_ as catalyst afforded the target TPIAnCN and TPIChCN with the yield of 96% and 92%, respectively. Their chemical structures were explicitly verified by standard methods (^1^H-NMR, ^13^C-NMR, high-resolution MS) (Figure S1).

**Figure 1:**
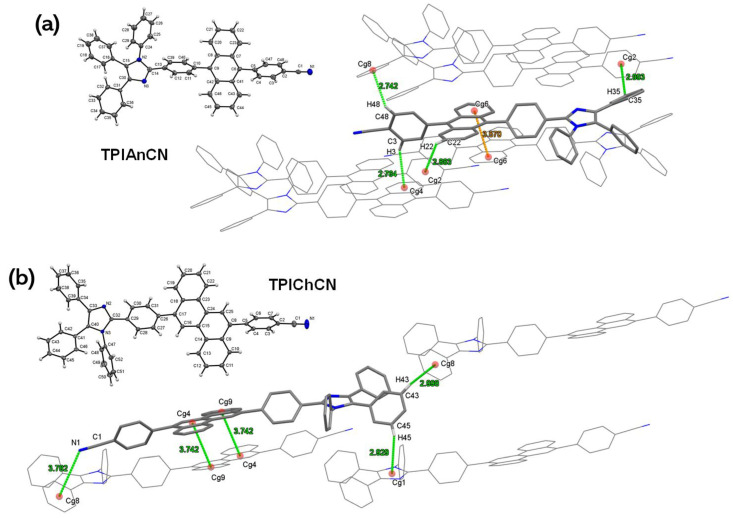
Single crystal structures (thermal ellipsoids at the 50% probability) and crystal packing of (**a**) TPIAnCN and (**b**) TPIChCN (C-H-π (green), π-π (orange), and *lp*-π (pink) interactions).

For a structural conformation and a deeper understanding of photophysical properties in the solid state of TPIAnCN and TPIChCN, an X-ray crystallographic analysis was performed. Their crystals for single-crystal X-ray diffraction (SC-XRD) studies were crystallized by using solvent and anti-solvent evaporation methods in a mixture of CHCl_3_/MeOH at room temperature. The structural refinement of TPIAnCN presented in the monoclinic crystal system with the space group of *P*21/c (a = 6.0526(4) Å, b = 14.6484(9) Å, c = 38.294(3) Å with β = 93.888 (3) Å). Whereas TPIChCN presented in the triclinic crystal system with the space group of *P*-1 (a = 10.0312(6) Å, b = 10.7769(7) Å, c = 18.8593(11) Å with β = 101.607 (2) Å and γ 107.107 (2) Å). The complete crystallographic information and molecular packing are given in Table S1 and Figures S2 and S3. As shown in Figure 1, both crystal structures showed their intermolecular interaction, mainly including CH-π and π-π interactions, fashioned face-to-face packing between aromatic planar units of anthracene for TPIAnCN and chrysene for TPIChCN. The interactions affected the fluorescent quenching phenomena in both solid powder and neat film by the continuous π–π interaction of anthracene and chrysene-cored structure (Figure 1), which caused a decrease in the PL quantum yield of the J-aggregation. Furthermore, a lone pair–π interaction (*lp*-π) was also found between the CN group and phenyl ring on the imidazole unit of TPIChCN, which caused the additional fluorescent quenching by electronic charge transfer [54]. To tackle both π-stacking and *lp*-π interaction which could hinder the OLED performance, a doped-layered OLED structure is introduced and studied.

**Figure 2:**
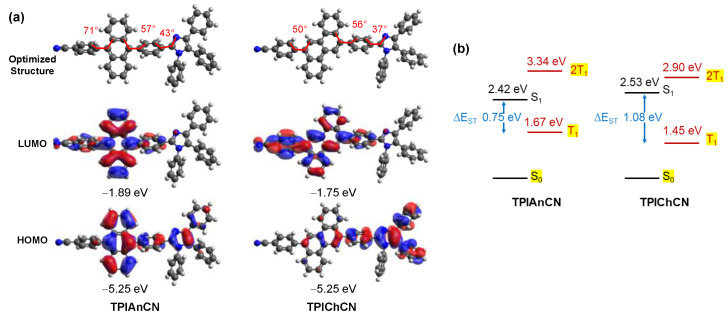
(**a**) The optimized structures, HOMO/LUMOs distribution, and energy levels calculated by B3LYP/6–31G(d,p) method in the gas phase. (**b**) The energy diagrams of the singlet (S) and triplet (T) excited states computed by TD-DFT B3LYP/6–31G(d,p) method.

For a deeper insight into the structural and electronic properties of TPIAnCN and TPIChCN, the theoretical calculations were performed using the B3LYP/6–31G(d,p) density functional theory (DFT) in CH_2_Cl_2_. As depicted in Figure 2a, their optimized structures showed a twisting conformation with the angles between anthracene and adjacent phenyl rings (71° and 57°) being larger than those of chrysene (50° and 56°). Hence, such highly twisted geometry would disrupt the molecular packing in the solid state to some degree, confining the fluorescence emission in the deep blue region. In frontier molecular orbitals, electrons in the highest occupied molecular orbital (HOMO) of TPIAnCN localized largely on the anthracene ring with a partial distribution on the TPI unit, whereas in the HOMO of TPIChAN, localization of electrons was observed mainly on the TPI moiety. On the other hand, the excited electrons in the lowest unoccupied molecular orbital (LUMO) of both molecules was delocalized over the conjugated backbones of 4-cyanophenyl anthracene for TPIAnCN and 4-cyanophenyl chrysene for TPIChAN. Additionally, the energy levels of both excited states (S and T) were measured using the time-dependent (TD)-DFT calculations using the B3LYP/6–31G(d,p) method. As illustrated in Figure 2b, the S_1_ and T_1_ excited energy levels of TPIAnCN and TPIChCN are calculated to be 2.42 and 2.53 eV, 1.67 eV, and 1.45 eV, respectively. As a result of their estimated ΔE_ST_ values of 0.75–1.45 eV, the difficulty is assured for both molecules to express the T_1_ to S_1_ reverse intersystem crossing (RISC) via the TADF mechanism. However, the two-triplet fusion energy levels (2T_1_) of the two molecules agree well with the principle of 2T_1_ > S_1_, verifying that the up-conversion of T_1_ into S_1_ via a TTA mechanism is achievable in both molecules [55,56].

**Figure 3:**
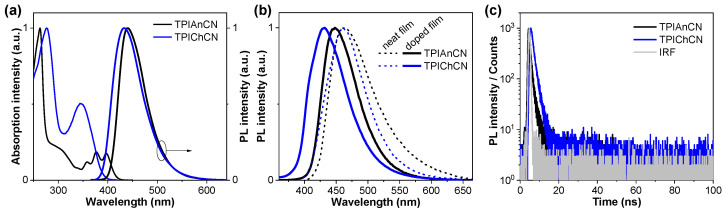
(**a**) UV-Vis absorption and PL spectra in toluene (~10^−5^ M). (**b**) PL spectra of the neat films and 5wt% doped in CBP films coated on fused silica substrates. (**c**) Transient PL spectra in neat films.

Figure 3 shows the optical properties of TPIAnCN and TPIChCN analyzed in solution (~10^−5^ M) and thin films (Figure 3 and Figure S4) and the key data are listed in Table 1. The UV-Vis absorption spectra in toluene of TPIAnCN and TPIChCN unveiled two obvious absorption bands around 260–270 nm originating from the aromatic rings [57] and 350–405 nm assigned to the π–π* transition of anthracene moiety and chrysene moiety of TPIAnCN and TPIChCN, respectively (Figure 3a) [50,58]. The optical bandgaps (E_g_^opt^) of TPIAnCN and TPIChCN were estimated from their absorption onsets to be 2.85 and 3.06 eV, respectively. In the solution, both molecules exhibited a strong fluorescence emission in a deep blue region. The photoluminescence (PL) spectra of TPIAnCN and TPIChCN were located at 441 and 432 nm, respectively, whereas their PL emission peaks in neat films showed a slight redshift to 463 and 462 nm for TPIAnCN and TPIChCN, owing to the enhancement of intermolecular interactions in the solid film, respectively (Figure 3b). The absolute PL quantum yields (Φ_PL_) measured by an integrating sphere of TPIAnCN and TPIChCN in toluene were evaluated to be 60% and 59%, respectively. On the one hand, in the neat film, their Φ_PL_ values of TPIAnCN and TPIChCN were dropped to 21% and 49%, respectively, signifying that intermolecular π–π interaction exists in the film state causing a fluorescence quenching. The high Φ_PL_ values of the molecules in film states could be restored by doping in 4’-bis(N-carbazolyl)-1,1′-biphenyl (CBP) film. With the optimum doping concentration of 5 wt%, the Φ_PL_ values of TPIAnCN and TPIChCN in film state were measured to be 71% and 73%, respectively. As shown in Figure 3b, the PL spectra of the doped films have shown slight blue-shift compared to the neat films with no emission peaks of the CBP host matrix being spotted, indicating a complete energy transfer from the CBP host to the dopants. A further study considering the PL lifetime measurements revealed that both compounds in neat film displayed mono-exponential decay profiles with the range of 0.96–1.41 ns (Figure 3c and Table 1), suggesting their PL emissions stem from the singlet excited state.

**Figure 4:**
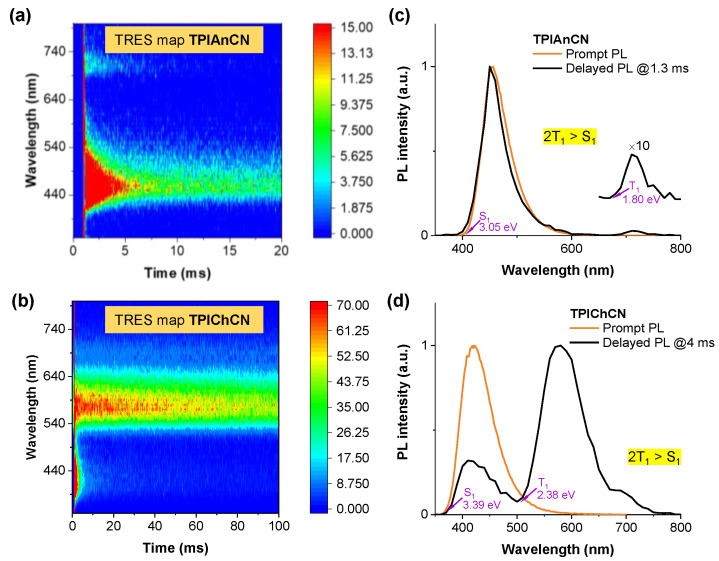
(**a**,**b**) TRES maps, and (**c**,**d**) integrated TRES slices of prompt and delayed PL spectra of TPIAnCN and TPIChCN 2 wt% doped poly(4-bromostyrene) films covered by EXCEVAL^TM^ film coated on fused silica substrates.

Additionally, TTA-induced delay fluorescence of TPIAnCN and TPIChCN were also investigated. The triplet energies of TPIAnCN and TPIChCN were measured using a room temperature triplet state spectroscopic measurement technique [59,60]. TPIAnCN and TPIChCN 2 wt% doped in poly(4-bromostyrene) (PBS) films covered by EXCEVALTM film were analyzed by time-resolved emission spectroscopy (TRES) (Figure 4). As depicted in Figure 4a,b, the TRES maps are composed of two-component emission maps in the ranges of 400–550 nm and 700–750 nm for TPIAnCN and 350–470 nm and 530–670 nm for TPIChCN. The integrated TRES slices of TPIAnCN (PL@1.3 ms) and TPIChCN (PL@4 ms) displayed two PL emission bands (Figure 4c,d). The PL bands at the low wavelength region matched well with their corresponding prompt PL emissions, verifying delayed PL from the S_1_ state. The PL bands at the longer wavelengths were assignable to their phosphorescence (Ph) emissions. Hence, the S_1_ state and T_1_ state energies were calculated from the onsets of those delayed PL and Ph spectra to be 3.05 and 1.80 eV for TPIAnCN, and 3.39 and 2.38 eV for TPIChCN, respectively [50,61]. TTA is considered to be the most plausible mechanism for the observed delayed PL among a few possible mechanisms. Both molecules could produce additional singlet excitons through the triplet fusion process since the energy levels fulfilled the conditions of 2T_1_ > S_1_ for TTA-based molecules. Furthermore, the energy gaps between S_1_ and T_1_ of greater than 1.25 eV would substantially limit the T_1_-to-S_1_ reverse intersystem crossing (RISC) process by way of the TADF mechanism [62].

**Figure 5:**
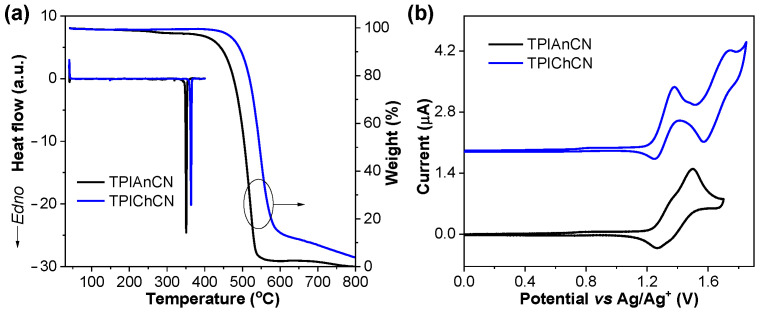
(**a**) TGA and DSC (2nd heating scan) plots analyzed at a heating rate of 10 °C min^−1^ under N_2_ flow. (**b**) Cyclic voltammograms recorded in dry CH_2_Cl_2_ containing *n-*Bu_4_NPF_6_ as a supporting electrolyte at a scan rate of 50 mV s^−1^ under an argon atmosphere.

The thermal properties of TPIAnCN and TPIChCN were examined by thermal gravimetric analysis (TGA) and differential scanning calorimetry (DSC) under a nitrogen flow. As shown in Figure 5a, both compounds exhibit high decomposition temperature (T_d_) TGA traces with the T_d_ at 5% weight loss over 500 °C (Table 1), suggesting high thermal stability. The 2nd heating DSC thermograms of TPIAnCN and TPIChCN display only sharp endothermic peaks corresponding to melting temperatures (T_m_) of 350, and 364 °C, respectively. No glass transition temperature was observed in both cases, implying that they are crystalline solids. The outstanding thermal stability of TPIAnCN and TPIChCN is critical to achieving a high device performance given that they can withstand high temperatures and will not easily decompose in the device fabrication process using thermal evaporation techniques. The electrochemical properties of TPIAnCN and TPIChCN were investigated using cyclic voltammetry (CV) at a scan rate of 50 mV s^−1^ in dry CH_2_Cl_2_ solution using 0.05 M of *n-*Bu_4_NPF_6_ as a supporting electrolyte. As described in Figure 5b, CV traces show two quasi-reversible oxidation waves (Table 1). The first oxidation process of both compounds appeared at the same half-wave potential (E_1/2_) of 1.32 V which could be associated with the oxidation of the TPI unit as observed in the DFT calculation results. The HOMO energy levels of TPIAnCN and TPIChCN in the thin film were determined by photoelectron yield spectroscopy (AC-2) in air to be −5.77 and −5.79 eV, respectively (Figure S5). The lowest unoccupied molecular orbital (LUMO) energy levels were calculated from the HOMO values and the optical band gaps (E_g_^opt^) by using the equation LUMO (eV) = HOMO + E_g_^opt^. The LUMO energy levels for TPIAnCN and TPIChCN were −2.92 and −2.73 eV, respectively.

**Figure 6:**
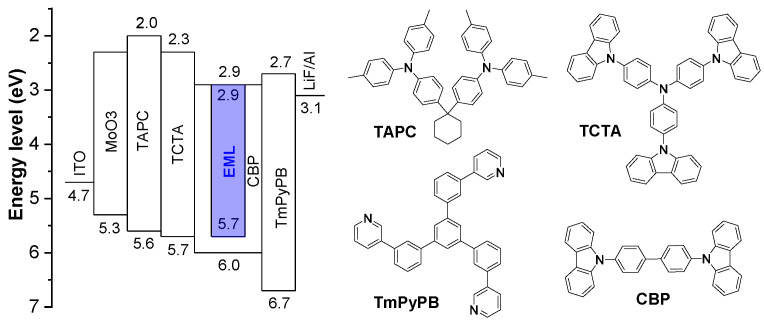
Energy diagram of the OLEDs and organic materials used in the devices.

**Figure 7:**
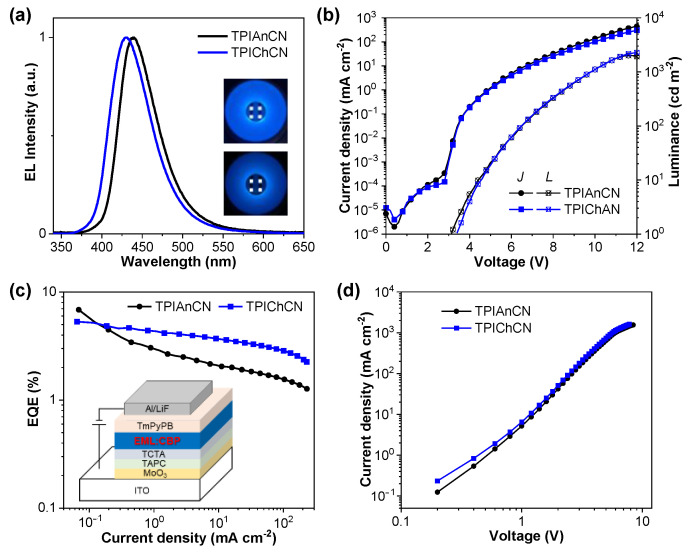
(**a**) Normalized electroluminescence (EL) spectra (insert: photographs of devices operated @ 8 V)), (**b**) Current density-voltage-luminance (*J-V-L*) plots and (**c**) EQE-current efficiency (*EQE-J*) plots of the OLEDs. (**d**) Current density-voltage (*J-V*) plots of the hole-only devices.

To evaluate the electroluminescence (EL) and TTA characteristics of TPIAnCN and TPIChCN as deep blue emitters, OLED devices with the structure of indium tin oxide (ITO)/molybdenum trioxide (MoO_3_) (10 nm)/1,1-bis[(di-4-tolylamino)phenyl]cyclohexane (TAPC) (80 nm)/tris(4-carbazoyl-9-ylphenyl)amine (TCTA) (5 nm)/emissive layer (EML) (20 nm)/1,3,5-tris[(3-pyridyl)-phen-3-yl]benzene) (TmPyPB) (40 nm)/lithium fluoride (LiF) (0.5 nm)/Al (100 nm) were fabricated and characterized as depicted in Figure 6. To achieve the optimal device EL performance, MoO_3_ as hole injection layer, TAPC as hole-transporting layer, TCTA as exciton blocking layer, TmPyPB as electron transporting layer and hole blocking layer, and LiF as electron injection layer were employed. TPIAnCN and TPIChCN 5wt% doped in CBP were used as EML. A combination of suitable hole mobility and HOMO level of TAPC (−5.50 eV) and suitable electron mobility and LUMO level of TmPyPB (−2.75 eV) will conceivably confine the exciton recombination zone width in EML. The characteristic curves and device data are shown in Figure 7 and Table 2. The OLEDs based on TPIAnCN and TPIChCN demonstrated intense blue emission color with the EL emission peaks at 438 and 431 nm with CIE coordinates of (0.15, 0.07) and (0.15, 0.05), respectively. Obviously, both devices delivered a deep blue light close to the HDTV standard blue color. The EL spectra of the two devices matched well with the PL spectra of the corresponding emitters in the thin films. Remarkably, the EL emissions were rather stable with no emission peaks of TAPC (374 nm), TCTA (390 nm) [63], CBP (390 nm) [64], or TmPyPB (471 nm) [64] being seen under the whole range of applied voltages (6–10 V) (Figure S6). This suggests an efficient charge injection and recombination in the EML, and excimer emission, as well as exciplex emissions at the interfaces of EML/TmPyPB and TCTA/EML, are effectively controlled. Both devices demonstrated low turn-on voltage (V_on_) of 3.2 and 3.4 V for TPIAnCN and TPIChCN, respectively, indicating effective charge injection and recombination in EML. Among the two, TPIAnCN-based OLED exhibited the highest EL performance with a maximum external quantum efficiency (EQE_max_) of 6.84%, whereas TPIChCN-based OLED showed a slightly lower EQE_max_ of 4.28%. However, in terms of color purity, the device based on TPIChCN with CIE coordinates of (0.15, 0.05) was in deeper blue emission color than TPIAnCN-based OLED. This suggests that the chrysene core is a deeper blue-emitting unit than the anthracene core.

**Figure 8:**
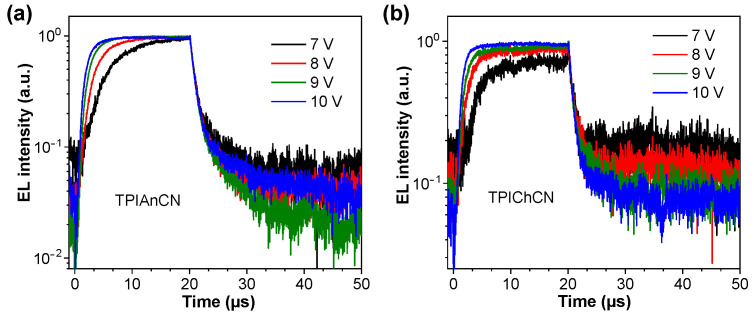
Transient EL decay profiles of the OLEDs at different applied voltages. (**a**) TPIAnCN and (**b**) TPIChCN.

To reveal the EL mechanism in these devices, transient EL measurements were performed. As shown in Figure 8, two components of prompt EL decay from rapid emission of S_1_ and delayed EL from the TTA channel are covered in the transient EL profiles of the two OLEDs. The delayed component ratio slightly decreased when the driving voltages increased indicating the presence of the TTA emission. The TTA process is extremely effective at a low driving voltage since this can create rich delayed components, giving rise to the improvement in the device performance. However, as the driving voltages increased, the delayed components decreased because the triplet excitons are quenched, resulting in decreased EQE [48].

To further realize the emitter’s behavior in the device, hole mobilities of the EMLs were estimated using the space-charge-limited current (SCLC) measurements of the hole-only devices (HOD) [65]. The hole-only devices were fabricated with the structure of ITO/MoO_3_ (10 nm)/EML (TPIAnCN and TPIChCN 5 wt% in CBP) (100 nm)/MoO_3_ (10 nm)/Al (100 nm). The current density-voltage (*J–V*) plots of HODs are shown in Figure 7d. Accordingly, TPIAnCN and TPIChCN emitters possess a relatively high and fast current with hole mobilities of 2.02 × 10^−4^ and 1.84 × 10^−4^ cm^2^ V^−1^s^−1^, respectively. Such high hole mobility could contribute to widening the recombination zone in the EML, resulting in a longer device lifetime as well as lower driving voltages. Consequently, the superior EL performance of the TPIAnCN-based OLED could be ascribed to a combination of a high thin film Φ_PL_, high hole mobility, and suitable HOMO/LUMO levels of the TPIAnCN emitter.

Furthermore, the singlet exciton utilization efficiency (η_s_) was calculated following the equation of EQE = η_out_ × η_rec_ × η_s_ × Φ_PL_, where the light outcoupling efficiency (η_out_) is 0.2 for glass substrate, and charge recombination efficiency (η_rec_) is estimated to be 1 [66]. As a result of the Φ_PL_ values of 71% for TPIAnCN emitter and 73% for TPIChCN emitter, the corresponding η_s_ values of TPIAnCN and TPIChCN-based devices were estimated to be 48% and 36%, respectively. These η_s_ surpass the statistical limit of 25% of traditional fluorescence emitters, verifying that both TPIAnCN and TPIChCN are TTA emitters. Therefore, the superb EL performance of both OLEDs could be ascribed to a combination of a high fluorescence feature and good hole-transporting property of the emitters as well as the TTA process in the device, in which all these properties are instigated by π-interactions of the polyaromatic rings (anthracene and chrysene) in the molecule in solid-state.

## 3. Conclusions

In summary, new triplet-triplet annihilation (TTA)-based deep blue emitters (TPIAnCN and TPIChCN) were designed and characterized. They demonstrated deep blue emission with peaks of around 430–440 nm with high fluorescence. TPIAnCN and TPIChCN were successfully fabricated as emitters in OLEDs. All devices displayed deep blue electroluminescence (EL) spectra with good color purity and CIE coordinates in high-definition television (HDTV) regions. In particular, TPIAnCN-based OLED reached the maximum EQE of 6.84% with CIE coordinates of (0.15, 0.07). The TPIChCN-based device represented one of the deepest blue-emitting TTA-OLEDs with CIE coordinates of (0.15, 0.05). This work not only comprehensively demonstrates the successful use of chrysene as an alternative building block to develop new TTA molecules for achieving high-performance deep blue TTA OLEDs, but also provides a novel design strategy and could potentially be beneficial for exploring high-performance blue OLEDs in the future.

## 4. Materials and Methods

All the reagents and solvents obtained from suppliers were used without further purification. The ^1^H- and ^13^C-NMR spectra were recorded using Bruker (Billerica, MA, USA) AVANCE III HD 600 MHz spectrometer with CDCl_3_ as a solvent. The high-resolution mass spectra were analyzed using APCI-TOF Bruker (Billerica, MA, USA) Compact mass spectrometer or Bruker (Billerica, MA, USA) Autoflex Speed^TM^ mass spectrometer. UV-Vis absorption spectra both in solution and thin film were measured using PerkinElmer (Waltham, MA, USA) model Lambda 1050 spectrophotometer. Photoluminescence spectra, lifetime, and TRES measurements both in solution and thin film were analyzed with an Edinburgh (Livingston, UK) FLS980 spectrophotometer. Absolute PL quantum yield was measured by the integrating sphere. Photoelectron spectroscopy (AC-2) was measured by Riken-Keiki (Itabashi, Tokyo, Japan) ultraviolet photoelectron spectrometer AC-2 in air. Thermogravimetric analysis (TGA) and differential scanning calorimetry (DSC) analysis were performed using a Rigaku (Akishima, Tokyo, Japan), model Thermoplus EV02 TG-DTA8122 and PerkinElmer ((Waltham, MA, USA), model DSC8500 with a heating rate of 10 °C min^−1^ under N_2_ gas flow. Cyclic voltammetry (CV) analyses were carried out using Metrohm (Ionenstrasse, Herisau, Switzerland) Autolab potentiostat PGSTAT 101 in CH_2_Cl_2_ under Ar atmosphere at a scan rate of 50 mV s^−1^ (platinum as a counter electrode, glassy carbon as a working electrode, Ag/AgCl as a reference electrode, *n-*Bu_4_NPF_6_ as a supporting electrolyte. Melting points were measured using a Krüss (Borsteler Chaussee, Hamburg, Germany) KSP1N melting point meter and were uncorrected. Single crystal X-ray diffraction (SC XRD) was collected using a Bruker (Billerica, MA, USA) D8 Venture spectrometer at 190 K (Mo K_α_ = 0.7107 Angstrom). The crystal refinement was calculated using APEX4, PLATON (100117), and OLEX2 software. All quantum chemical calculations were based on density functional theory (DFT) and performed with Gaussian 16 program package [67]. The ground state geometries, HOMO and LUMO distributions, and HOMO and LUMO energy levels were calculated by the B3LYP/6–31G(d,p) level of theory. The energy level of the singlet (S) excited and triplet (T) excited states were computed using time-dependent (TD)-DFT calculation with the B3LYP/6–31G(d,p) method.

The OLED devices with the structure of ITO/MoO_3_ (10 nm)/TAPC (80 nm)/TCTA (5 nm)/emissive layer (TPIAnCN and TPIChCN 5wt% doped in CBP (20 nm)/TmPyPB (40 nm)/LiF (0.5 nm)/Al (100 nm) were fabricated and characterized as follows. Indium tin oxide (ITO) glass substrates (12 Ω sq^−1^) were cleaned, dried in a vacuum oven under 120 °C for 1 h, and finally treated with UV-ozone for 30 min. The substrate was then transferred to a vacuum deposition system with a base pressure lower than 5 × 10^−5^ Pa. The inorganic layer of molybdenum trioxide (MoO_3_) was evaporated at a rate of 0.2 Å s^−1^. The organic layers of di-(4-(N,N-ditolyl-amino)-phenyl)cyclohexane (TAPC), tris(4-carbazoyl-9-ylphenyl)amine (TCTA), emissive layer (EML) (TPIAnCN and TPIChCN 5wt% doped 4,4′-bis(N-carbazolyl)-1,1′-biphenyl (CBP)) and (1,3,5-tri(m-pyridin-3-ylphenyl)benzene (TmPyPB) were evaporated at rates of 0.2–0.3 Å s^−1^. The cathode was completed through thermal deposition of lithium fluoride (LiF) at a deposition rate of 0.2 Å s^−1^, and then capped with aluminum through thermal deposition at a deposition rate of 0.5–1 Å s^−1^. Current density-voltage-luminance (*J–V–L*) characteristics were measured simultaneously using a Keithley (Beaverton, OR, USA) 2400 source meter and a Hamamatsu Photonics (Hamamatsu, Shizuoka, Japan) PMA-12 multi-channel analyzer. The absolute external quantum efficiency (EQE) of OLED devices was obtained by Hamamatsu Photonics (Hamamatsu, Shizuoka, Japan) C9920–12 External Quantum Efficiency Measurement System utilizing an integrating sphere. All the measurements were performed under an ambient atmosphere at room temperature. The transient electroluminescence (EL) data were acquired using an arbitrary function generator (AFG1022, Tektronix (Beaverton, OR, USA)) and a Si photodiode (OSD15-E, Centronic (Croydon, Surrey, UK)), connected to a variable-gain highspeed current amplifier (DHPCA100, Femto (Messtechnik GmbH., Berlin, Germany)). The OLED devices applied a positive pulse voltage with a pulse width of 20 μs and frequency of 1 kHz. To remove the partial delay emission from the recombination of trapped charges, the negative pulse voltage of –5.0 V was applied immediately after applying a positive pulse voltage. The delayed EL signals were collected using a mixed domain oscilloscope (500 MHz, MDO32, Tektronix (Beaverton, OR, USA)). Moreover, hole-only devices (HOD) were also fabricated by the configuration of ITO/MoO_3_ (10 nm)/TPIAnCN and TPIChCN 5wt% doped in CBP (80 nm)/MoO_3_ (10 nm)/Al (100 nm).

Synthesis of 4-(10-(4-(1,4,5-triphenyl-1H-imidazol-2-yl)phenyl)anthracen-9-yl)benzonitrile (TPIAnCN): A mixture of 3 (200 mg, 0.28 mmol) and 2 (364 mg, 0.36 mmol), 10% K_2_CO_3_ (aq) (5 mL) and Pd(PPh_3_)_4_ (34 mg, 5 mol%) in THF (30 mL) was degassed with nitrogen for 10 min. The reaction mixture was heated at reflux under nitrogen for 18 h. After cooling, water was added, and the mixture was extracted by CH_2_Cl_2_ (3 × 50 mL). The combined organic layer was washed with water (50 mL), brine solution (50 mL), dried over anhydrous Na_2_SO_4_, filtered, and concentrated in vacuo to dryness. The crude product was purified by column chromatography on silica gel eluting with CH_2_Cl_2_:hexane (2:3) to give slight yellow solids (350 mg, 96%). M.p. = 345–346 °C; ^1^H-NMR (600 MHz, CDCl_3_) δ 7.91 (2H, d, *J* = 8.1 Hz, Ar-H), 7.69 (6H, t, *J =* 7.8 Hz, Ar-H), 7.61 (2H, d, *J* = 8.1 Hz, Ar-H), 7.54–7.50 (2H, m, Ar-H), 7.39–7.34 (9H, m, Ar-H), 7.31–7.26 (6H, m, Ar-H), 7.24–7.19 (5H, m, Ar-H). ^13^C-NMR (151 MHz, CDCl_3_) δ 146.65, 144.49, 138.62, 138.51, 137.64, 137.25, 134.66, 134.45, 132.31, 132.29, 131.21, 131.10, 130.68, 130.12, 129.68, 129.38, 129.19, 128.90, 128.59, 128.50, 128.42, 128.21, 128.09, 127.40, 127.14, 126.71, 126.04, 125.74, 125.26, 118.89, 111.68, 77.23, 77.02, 76.81. HRMS APCI (*m*/*z*): calcd for C_48_H_31_N_3_: 649.2518, found: 650.2503 [MH]^+^.

Synthesis of 4-(12-(4-(1,4,5-triphenyl-1H-imidazol-2-yl)phenyl)chrysen-6-yl)benzonitrile (TPIChCN): A mixture of 4 (200 mg, 0.50 mmol) and 2 (500 mg, 1.0 mmol), 10% K_2_CO_3_ (aq) (5 mL), and Pd(PPh_3_)_4_ (29 mg, 5 mol%) in THF (45 mL) was degassed with nitrogen for 10 min. The reaction mixture was heated at reflux under nitrogen for 18 h. After cooling, water was added and the mixture was extracted by CH_2_Cl_2_ (3 × 50 mL). The combined organic layer was washed with water (50 mL), brine solution (50 mL), dried over anhydrous Na_2_SO_4_, filtered, and concentrated in vacuo to dryness. The crude product was purified by column chromatography on silica gel eluting with CH_2_Cl_2_:hexane (2:3) to give white solids (320 mg, 92%). M.p. = 351–353 °C; ^1^H-NMR (600 MHz, CDCl_3_) δ 8.83 (1H, d, *J* = 8.5 Hz, Ar-H), 8.79 (1H, d, *J* = 8.5 Hz, Ar-H), 8.62 (2H, d, *J* = 8.3 Hz, Ar-H), 8.01 (1H, d, *J* = 8.3 Hz, Ar-H), 7.88–7.84 (3H, m, Ar-H), 7.77–7.62 (8H, m, Ar-H), 7.58 (2H, q, *J* = 7.4 Hz, Ar-H), 7.52 (2H, d, *J* = 7.8 Hz, Ar-H), 7.37–7.31 (3H, m, Ar-H), 7.30–7.15 (10H, m, Ar-H); ^13^C-NMR (151 MHz, CDCl_3_) 146.6, 146.2, 139.5, 138.5, 137.3, 134.5, 132.3, 131.2, 131.2, 131.0, 130.9, 130.8, 130.3, 130.0, 129.9, 129.2, 128.8, 128.6, 128.5, 128.4, 128.2, 128.0, 127.8, 127.4, 127.1, 127.0, 126.9, 126.7, 126.2, 123.7, 123.3, 122.4, 121.9, 118.9, 111.4; HRMS MALDI-TOF (*m*/*z*): calcd for C_52_H_33_N_3_: 699.2674, found: 699.2675 [M]^+^.

## Figures and Tables

**Table 1:** Key optical and physical data of TPIAnCN and TPIChCN in solution and thin films.

Compd	λ_abs_ (log(ε)) ^a^ (nm, M^−1^ cm^−1^)	λ_PL_ (nm) sol ^a^/Film ^b^/ dfilm ^d^	Φ_PL_ (%) ^c^ Sol ^a^/Film ^b^/ dfilm ^d^	τ (ns) ^e^ sol ^a^/Film ^b^	T_m_/T_d_ (°C) ^f^	E_1/2_ vs. Ag/Ag^+^ (V) ^g^	E_g_^opt^ (eV) ^h^	HOMO/LUMO (eV) ^i^
TPIAnCN	263 (5.51), 357 (4.59), 376 (4.77), 396 (4.75)	441/463/446	60/21/71	2.89/0.96	350/540	1.32, 1.65	2.85	−5.77/−2.92
TPIChCN	276 (5.07), 345 (4.77)	432/462/432	59/49/73	1.39/1.41	364/568	1.32, 1.43	3.06	−5.79/2.73

^a^ Analyzed in toluene solution. ^b^ Analyzed as thin films coated on fused silica substrates. ^c^ Absolute PL quantum yield measured with an integrating sphere. ^d^ Analyzed as 5wt% doped CBP films. ^e^ Transient PL decay time. ^f^ Determined by TGA and DSC at a heating rate of 10 °C min^−1^ under N_2_ flow. ^g^ Obtained from CV measurement at a scan rate of 50 mV s^−1^. ^h^ Calculated from the onset of absorption spectra of thin films: E_g_^opt^ = 1240/λ_onset_. ^i^ HOMO obtained from AC-2 of the neat film and LUMO (eV) = HOMO + E_g_^opt^.

**Table 2:** Key performance parameters of the fabricated OLEDs using TPIAnCN and TPIChCN as emitters.

Emitter ^a^	V_on_ (V) ^b^	λ_EL_ (nm) ^c^	FWHM (nm) ^d^	L_max_ (cd m^−2^) ^e^	J_max _ (mA cm^−2^) ^f^	EQE_max_/EQE_500_ (%) ^g^	CIE (x, y) ^h^	η_s_ (%) ^i^
TPIAnCN	3.2	438	55	2016	436	6.84/1.72	(0.15, 0.07)	48%
TPIChCN	3.4	429	58	2282	307	5.31/3.38	(0.15, 0.05)	36%

^a^ The uniform device structure: ITO/MoO_3_ (10 nm)/TAPC (80 nm)/TCTA (5 nm)/emitter 5 wt% in CBP (20 nm)/TmPyPB (40 nm)/LiF (0.05 nm)/Al (100 nm). ^b^ Turn on voltage at 1 cd m^−2^. ^c^ EL emission peak. ^d^ Full width half maximum. ^e^ Maximum luminance. ^f^ Maximum current density. ^g^ External quantum efficiencies at maximum and 500 cd m^−2^. ^h^ Commission Internationale de I’Eclairage coordinate. ^i^ Singlet utilization efficiency.

## Data Availability

The data presented in this study are available in the article.

## Supplementary Materials

The following supporting information can be downloaded at: https://www.mdpi.com/article/10.3390/molecules27248923/s1, Materials synthesis and characterization. Figure S1: Copies of 1H/13C- NMR spectra and HRMS mass spectra. Table S1: Crystallographic data of TPIAnCN and TPIChCN. Figure S2: Crystal packing of (top) TPIAnCN and (bottom) TPIChCN along [100], [010] and [001] directions. Figure S3: Packing structure of (top) TPIAnCN and (bottom) TPIChCN along [110] and [210] directions. Figure S4: PL spectra in different solvents. Figure S5: AC-2 plots. Figure S6: EL spectra under different applied voltages.

## Sample Availability

Samples of the compounds are not available from the authors.
