# Supplementary material for: Deep-Blue Triplet–Triplet Annihilation Organic Light-Emitting Diode (CIEy ≈ 0.05) Using Tetraphenylimidazole and Benzonitrile Functionalized Anthracene/Chrysene Emitters
Source: Molecules. 2022 Dec 15;27(24):8923. doi: 10.3390/molecules27248923 (PMC9787557; doi:10.3390/molecules27248923)
Supplement: Supplementary file 1 [file molecules-27-08923-s001.zip › molecules-2096747-supplementary.pdf]

Supporting Information

Materials synthesis and characterization

2-(4-Bromophenyl)-1,4,5-triphenyl-2,5-dihydro-1H-imidazole (1): A mixture of benzil (1.5 g, 7.10 mmol), 4-bromobenzaldehyde (1.32 g, 7.10 mmol) was dissolved in acetic acid (30 mL), and stirred at room temperature. Aniline (0.98 ml, 10.70 mmol) was added dropwise and ammonium acetate (2.75 g, 35.70 mmol) was then added to the reaction mixture, followed by heat to 110°C for 12 hours. After completion, the reaction was allowed to room temperature and pour into an ice bath. The crude solid was filtered and washed with water. The filtered solid was dissolved in dichloromethane, washed with water (4 x 30 ml), dried (Na_2SO_4), and concentrated *in vacuo*. The product was purified by recrystallization in dichloromethane/methanol to give light brown solids (2.30 g, 74%). ^{1}H-NMR (600 MHz, $CDCl_3$) δ 7.60-7.55 (2H, m, Ar-*H*), 7.39-7.35 (2H, m, Ar-*H*), 7.32 - 7.17 (11H, m, Ar-*H*), 7.12 (2H, d, *J* = 6.8 Hz, Ar-*H*), 7.04(2H, d, *J* = 6.8 Hz, Ar-*H*); ^{13}C-NMR (151 MHz, $CDCl_3$) δ 145.8, 138.5, 136.9, 134.3, 131.3, 131.2, 131.1, 130.5, 130.3, 129.5, 129.2, 128.5, 128.4, 128.2, 128.1, 127.4, 126.7, 122.6. HRMS APCI (*m/z*): calcd for $C_{48}H_{31}N_3$: 452.0711, found: 453.0672 $[MH]^+$.

1,4,5-Triphenyl-2-(4-(4,4,5,5-tetramethyl-1,3,2-dioxaborolan-2-yl)phenyl)-2,5-dihydro-1H-imidazole (2): A mixture of **1** (2.2 mmol), bispinacolatodiboron (1.7 g, 7 mmol), and potassium acetate (2.7g, 27.0 mmol) was suspended in dried toluene (40 mL) under a nitrogen atmosphere. Bis(triphenylphosphine)palladium(II) dichloride $Pd(PPh_3)_2Cl_2$ (77.2 mg, 0.11 mmol) was added and degassed for 10 minutes. The reaction mixture was stirred for 12 hours under reflux, cooled to room temperature and water was added. The resulting mixture was extracted with dichloromethane (4 x 70 ml), washed the organic layer with brine, dried over Na_2SO_4, and concentrated *in vacuo*. The product was purified by column chromatography on silica gel eluting with hexane:dichloromethane (6:4) to give white solids (1.13 g, 90%). ^{1}H-NMR (600 MHz, $CDCl_3$) δ 7.67 (2H, d, *J* = 8.0 Hz, Ar-*H*), 7.60 (2H, d, *J* = 7.4 Hz, Ar-*H*), 7.43 (2H, d, *J* = 8.0 Hz, Ar-*H*), 7.29-7.17 (7H m, Ar-*H*), 7.14 (2H, d, *J* = 8.0 Hz, Ar-*H*), 7.04 (2H, d, *J* = 7.0 Hz, Ar-*H*), 1.32 (12H, s, $(CH_3)_4$); ^{13}C-NMR (151 MHz, $CDCl_3$) δ 146.7, 138.5, 137.2, 134.4, 134.4, 133.0, 131.2, 131.1, 130.6, 129.1, 128.4, 128.3, 128.3, 128.1, 128.0, 127.4, 126.6, 83.9, 24.9. HRMS APCI (*m/z*): calcd for $C_{48}H_{31}N_3$: 498.2479, found: 498.2422 $[M]^+$.

4-(10-Bromoanthracen-9-yl)benzonitrile (3): A mixture of 9,10-dibromoanthracene (200 mg, 1.36 mmol) and 4-cyanophenylboronic acid (1.4 g, 4.08 mmol) was dissolved in dried THF (30 mL) under a nitrogen atmosphere. 10% K_2CO_3 (aq) (10 mL) was added and stirred at ambient temperature. Tetrakris(triphenylphosphene)palladium(0) $Pd(PPh_3)_4$ (78.6 mg, 5 mol%) was then added and degassed the reaction mixture for 10 minutes. The reaction mixture was heated to reflux. After 18 h the reaction was stopped, cooled to room temperature, and water was added to the reaction mixture. The resulting solution was extracted by dichloromethane (3 x 50 mL) and the organic layer was washed with brine (3 x 20 mL), dried (Na_2SO_4), and concentrated *in vacuo*. Purification by column chromatography on silica gel eluting with gradient hexane:dichloromethane from 100% to 50% to give yellow solids (318 mg, 65%). ^{1}H-NMR (600

MHz, $CDCl_3$) δ 8.59 (2H, dt, *J* = 8.9, 0.9 Hz, Ar-*H*), 7.70–7.64 (4H, m, Ar-*H*), 7.62 (2H, dt, *J* = 8.8, 0.9 Hz, Ar-*H*), 7.58 (2H, ddd, *J* = 8.9, 6.5, 1.2 Hz, Ar-*H*), 7.37 (2H, ddd, *J* = 8.7, 6.5, 1.1 Hz, Ar-*H*), 7.34 (3H, dd, *J* = 5.0, 1.9 Hz, Ar-*H*), 7.31–7.24 (8H, m, Ar-*H*), 7.23–7.17 (5H, m, Ar-*H*); ^{13}C NMR (151 MHz, $CDCl_3$) δ 146.6, 138.5, 138.3, 137.2, 137.2, 134.4, 131.2, 131.0, 130.9, 130.7, 130.2, 130.1, 129.2, 128.6, 128.5, 128.4, 128.2, 128.1, 127.9, 127.4, 127.3, 126.9, 126.7, 125.6, 122.8. HRMS APCI (*m/z*): calcd for $C_{48}H_{31}N_3$: 357.0153, found: 358.0166 $[MH]^+$.

4-(12-Bromochrysen-6-yl)benzonitrile (4): A mixture of 6,12-Dibromochrysene (700 mg, 1.80 mmol) and 4-cyanophenylboronic acid (88 mg, 0.6 mmol) was dissolved in dried THF (60 mL) under a nitrogen atmosphere. 10% K_2CO_3 (aq) (10 ml) was added and stirred at ambient temperature. Tetrakris(triphenylphosphene)palladium(0) $Pd(PPh_3)_4$ (35 mg, 5 mol%) was then added and degassed the reaction mixture for 10 minutes. The reaction mixture was heated to reflux. After 18h the reaction was stopped, cooled to room temperature, and water was added to the reaction mixture. The resulting solution was extracted by dichloromethane (3 x 50 mL) and the organic layer was washed with brine (3 x 20 mL), dried (Na_2SO_4), and concentrated *in vacuo.* Purification by column chromatography on silica gel eluting with gradient hexane:dichloromethane from 100% to 50% to give yellow solids (147.6 mg, 60%). ^1H-NMR (600 MHz, $CDCl_3$) δ 9.09 (1H, s, Ar-*H*), 8.80 (1H, d, *J* = 8.4 Hz, Ar-*H*), 8.78–8.73 (1H, m, Ar-*H*), 8.56 (1H, s, Ar-*H*), 8.50–8.45 (1H, m, Ar-*H*), 7.92–7.84 (m, 3H), 7.81–7.72 (m, 5H), 7.62 (1H, ddd, *J* = 8.1, 6.8, 1.2 Hz, Ar-*H*); ^{13}C-NMR (151 MHz, $CDCl_3$) δ 145.9, 137.8, 132.3, 131.4, 130.9, 130.8, 130.4, 130.0, 128.7, 128.3, 127.8, 127.8, 127.3, 127.2, 127.1, 126.2, 125.3, 123.6, 123.5, 123.3, 122.3, 118.8, 111.6. HRMS APCI (*m/z*): calcd for $C_{48}H_{31}N_3$: 409.0289, found: 409.9745 $[MH]^+$.

Figure S1. Copies of ¹H/¹³C- NMR spectra and HRMS mass spectra

2-(4-Bromophenyl)-1,4,5-triphenyl-2,5-dihydro-1H-imidazole (1)

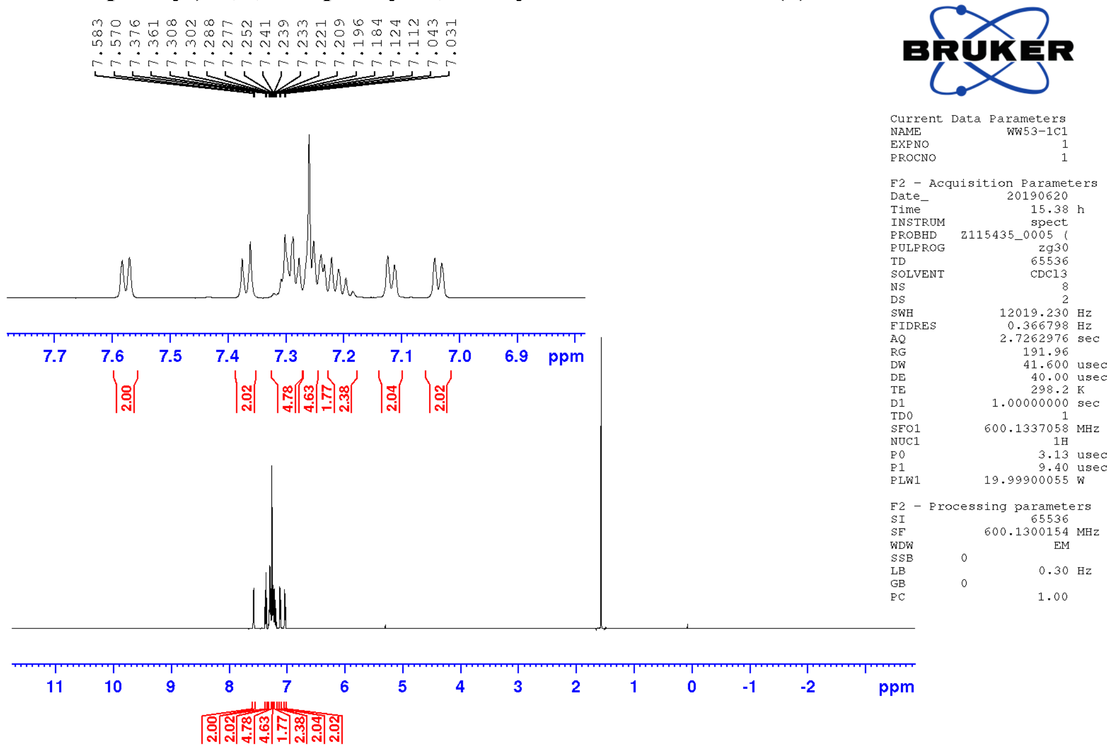

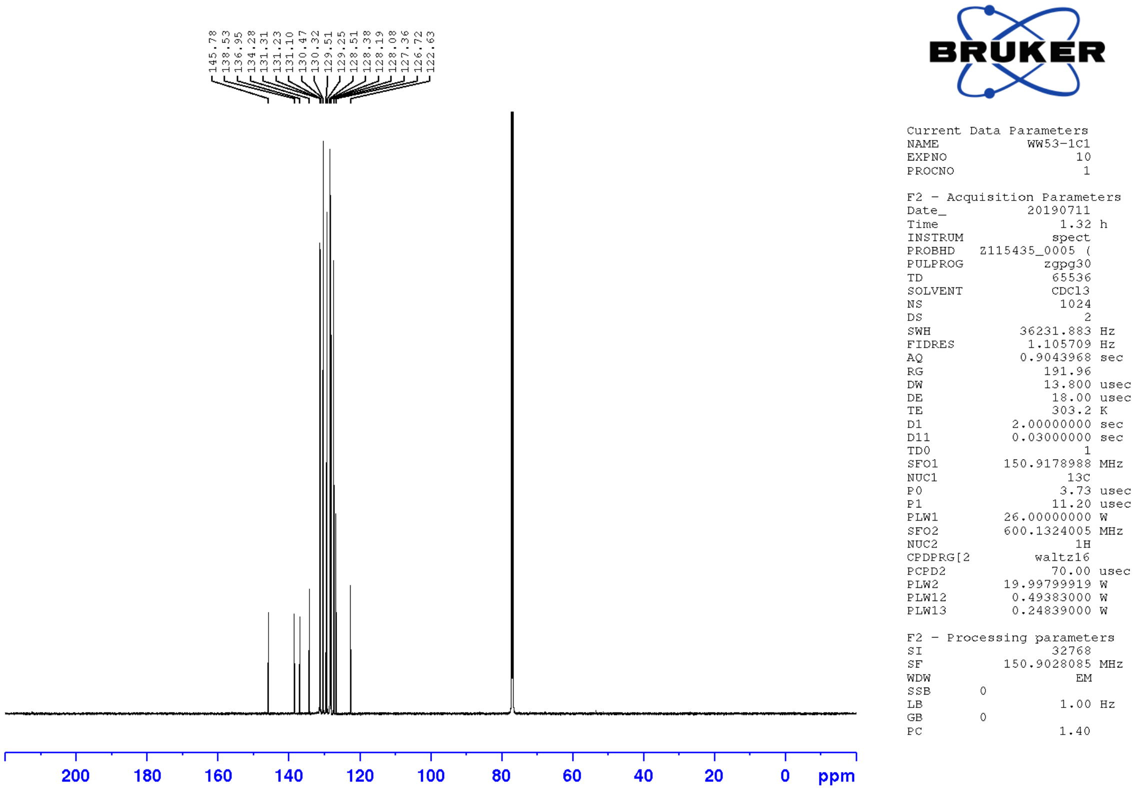

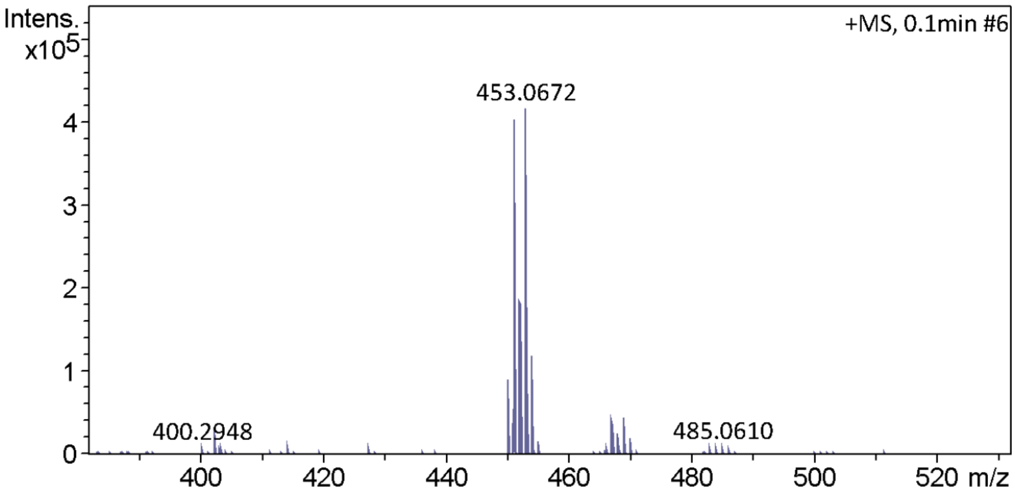

1,4,5-Triphenyl-2-(4-(4,4,5,5-tetramethyl-1,3,2-dioxaborolan-2-yl)phenyl)-2,5-dihydro-1H-imidazole (2)

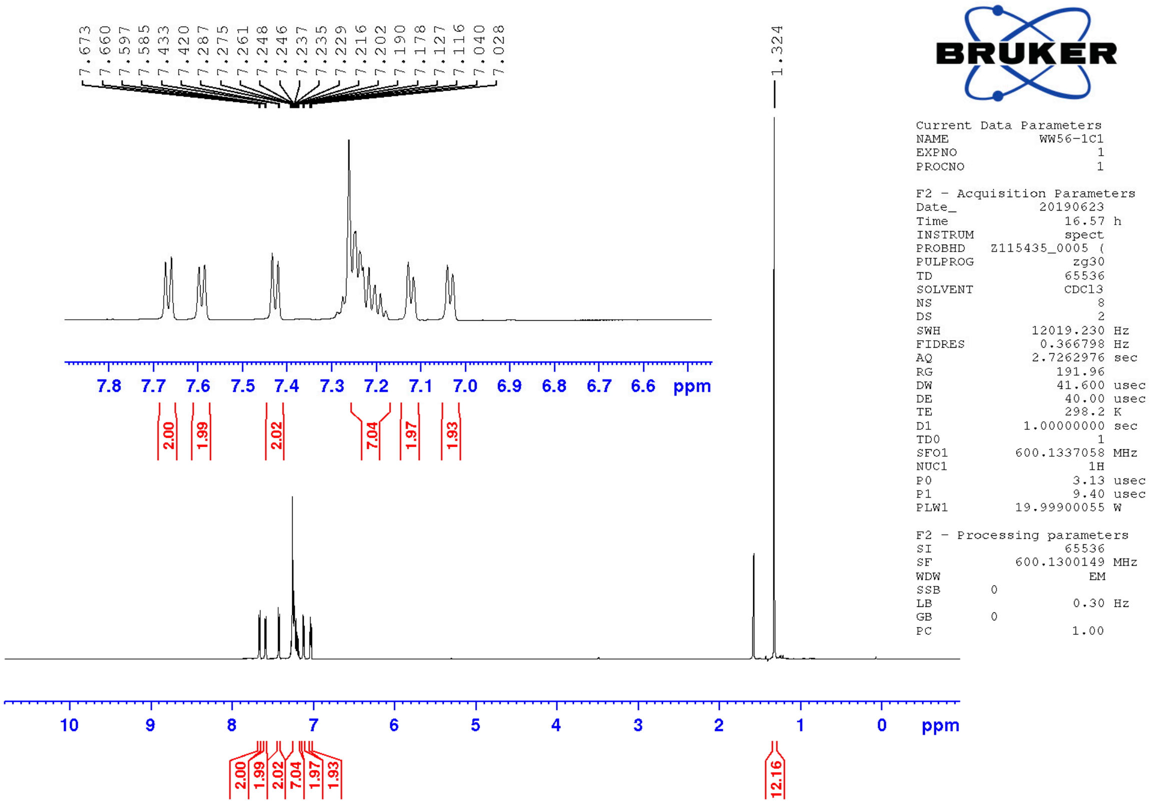

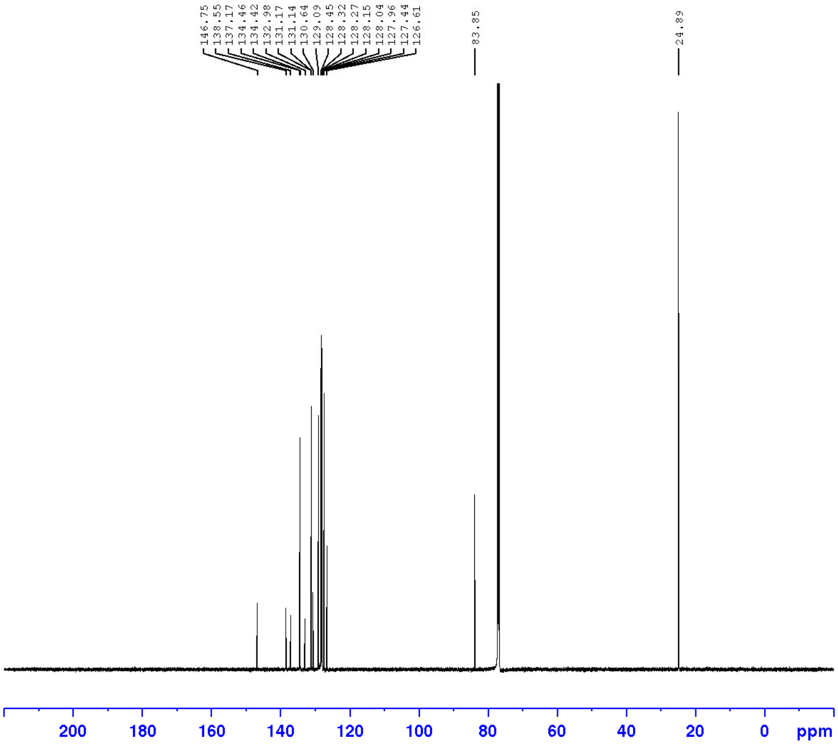

Current Data Parameters
NAME WW56-1C1
EXPNO 10
PROCNO 1

F2 - Acquisition Parameters
Date_ 20190711
Time 2.26 h
INSTRUM spect
PROBHD Z115435_0005 (
PULPROG zgpg30
TD 65536
SOLVENT CDCl3
NS 1024
DS 2
SWH 36231.883 Hz
FIDRES 1.105709 Hz
AQ 0.9043968 sec
RG 191.96
DW 13.800 usec
DE 18.00 usec
TE 303.2 K
D1 2.00000000 sec
D11 0.03000000 sec
TD0 1
SFO1 150.9178988 MHz
NUC1 13C
P0 3.73 usec
P1 11.20 usec
PLW1 26.00000000 W
SFO2 600.1324005 MHz
NUC2 1H
CPDPRG[2 waltz16
PCPD2 70.00 usec
PLW2 19.99799919 W
PLW12 0.49383000 W
PLW13 0.24839000 W

F2 - Processing parameters
SI 32768
SF 150.9028085 MHz
WDW EM
SSB 0
LB 1.00 Hz
GB 0
PC 1.40

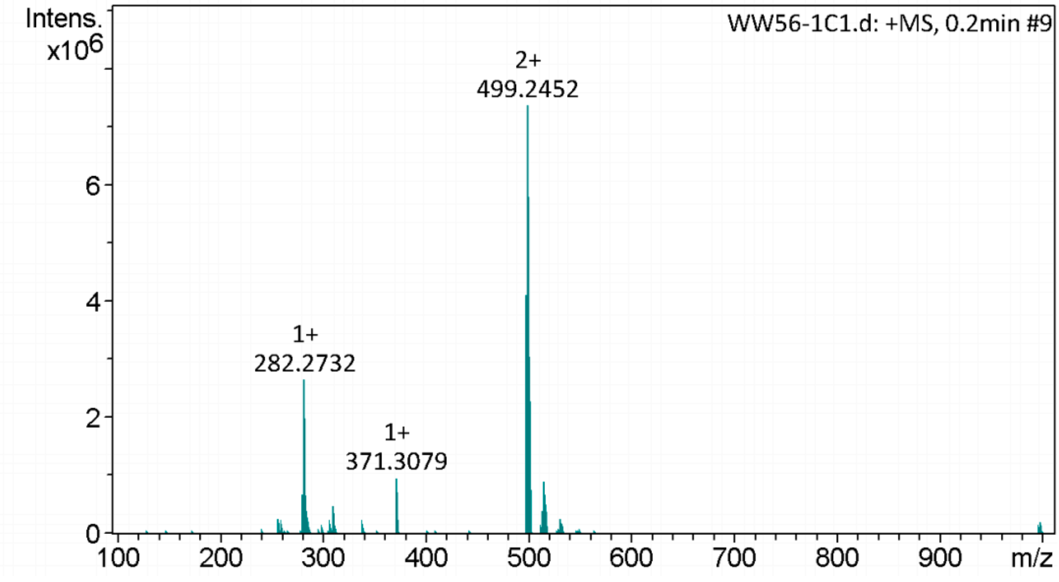

4-(10-Bromoanthracen-9-yl)benzonitrile (3)

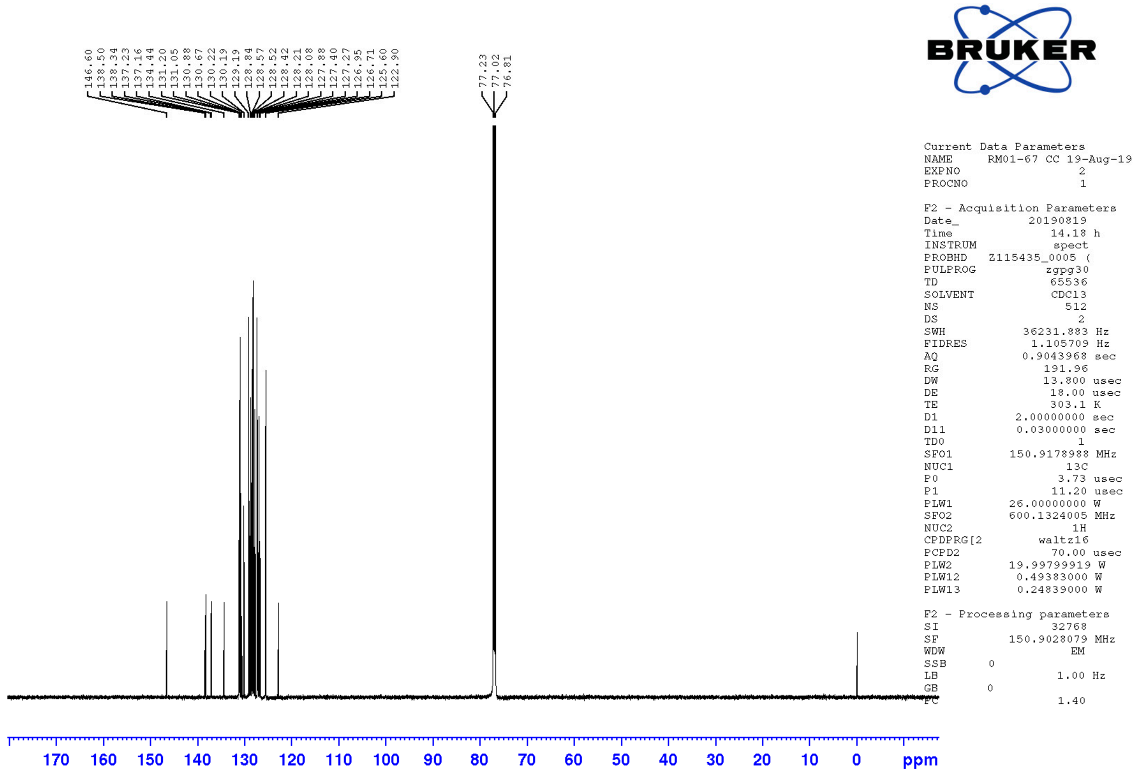

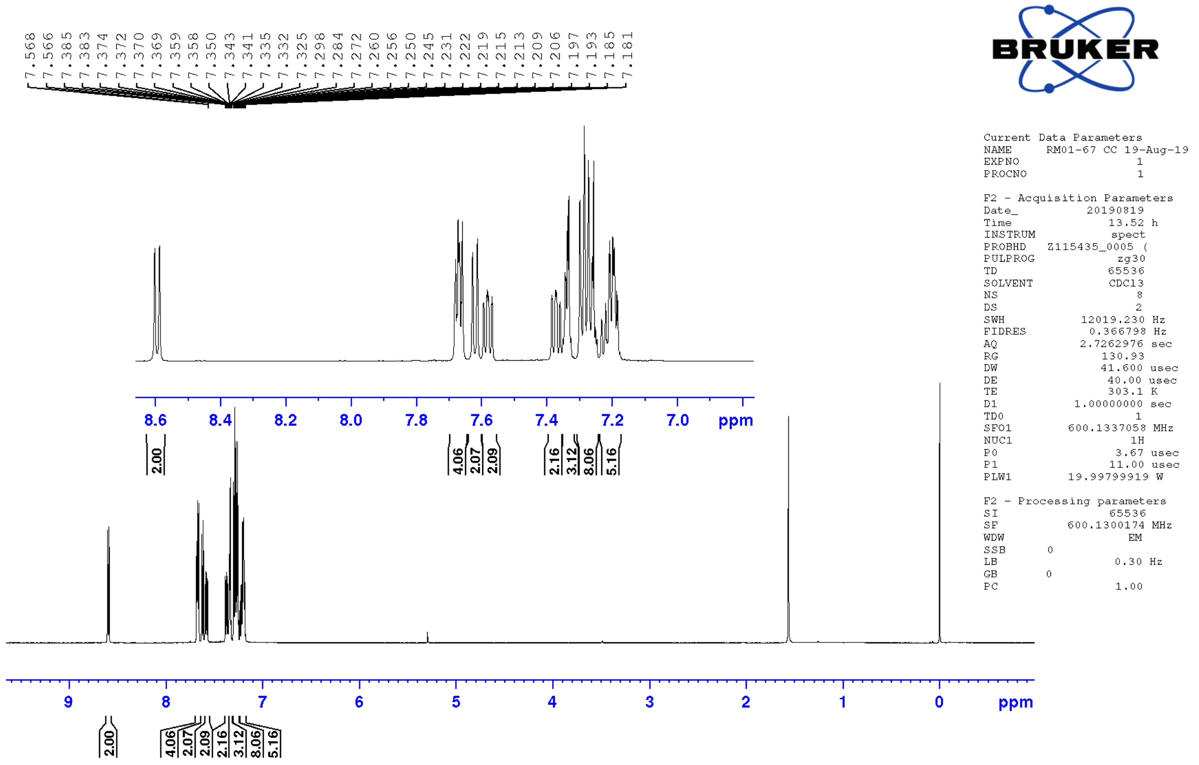

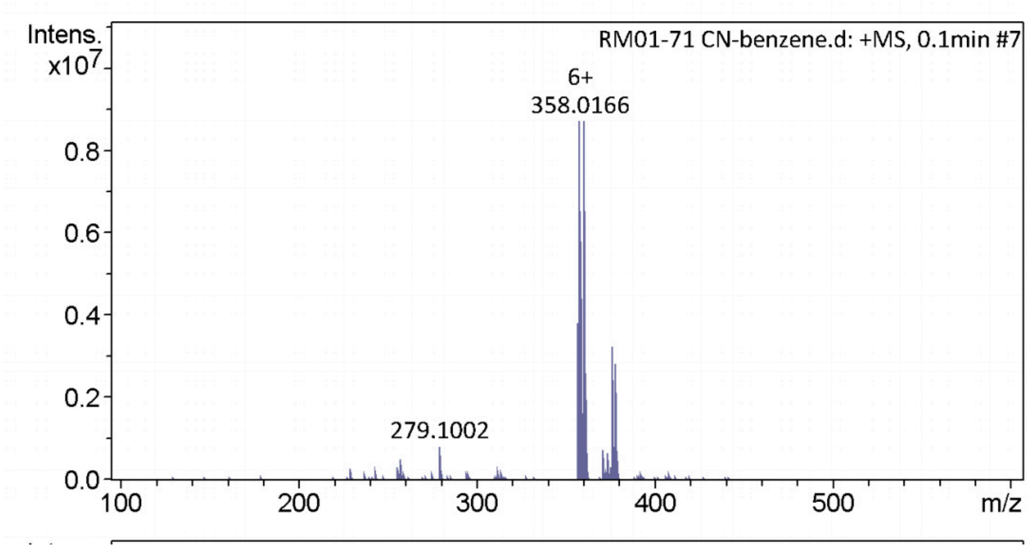

4-(12-Bromochrysen-6-yl)benzonitrile (4)

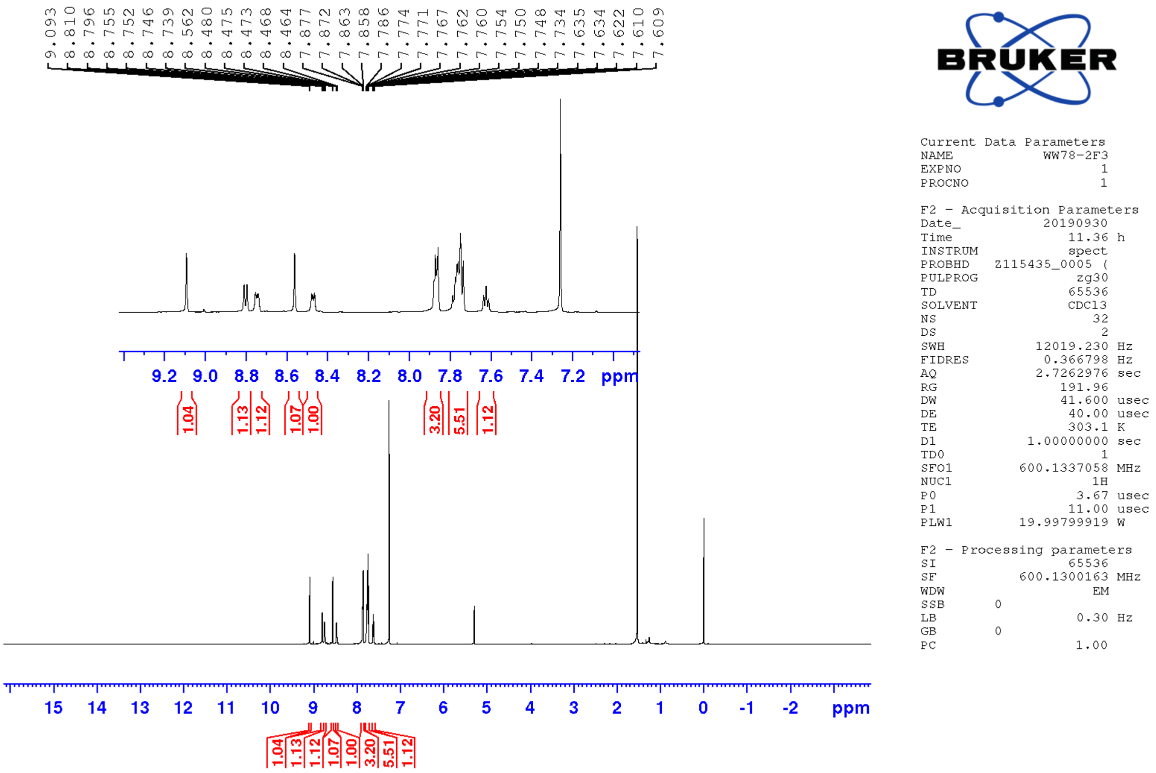

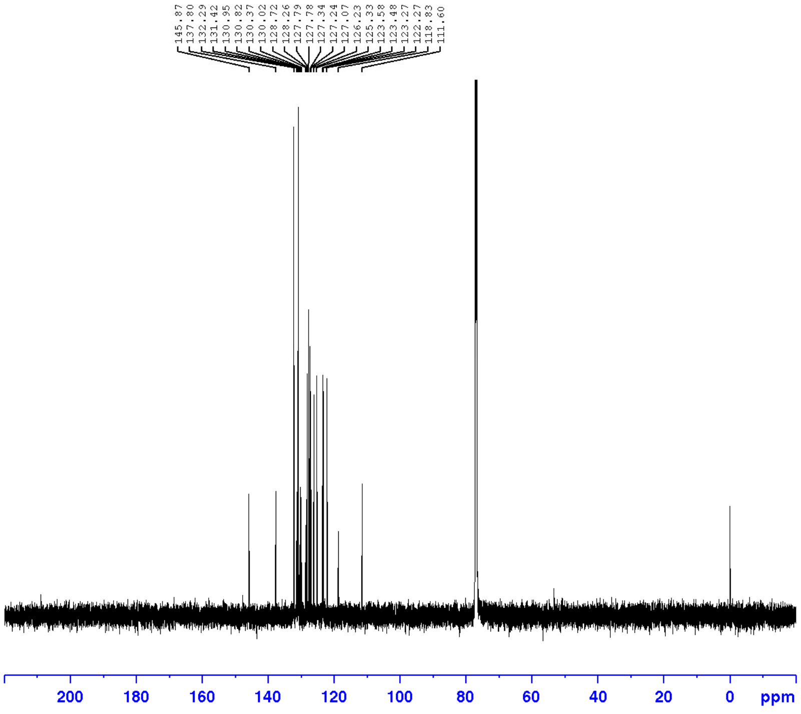


```
Current Data Parameters
NAME            WW78-2F3
EXPNO                 10
PROCNO                 1

F2 - Acquisition Parameters
Date_           20190930
Time               15.59 h
INSTRUM            spect
PROBHD   Z115435_0005 (
PULPROG          zgpg30
TD                 65536
SOLVENT            CDCl3
NS                   256
DS                     2
SWH            36231.883 Hz
FIDRES          1.105709 Hz
AQ             0.9043968 sec
RG                191.96
DW                13.800 usec
DE                 18.00 usec
TE                 303.1 K
D1            2.00000000 sec
D11           0.03000000 sec
TD0                    1
SFO1         150.9178988 MHz
NUC1                 13C
P0                  3.60 usec
P1                 10.80 usec
PLW1         26.00000000 W
SFO2         600.1324005 MHz
NUC2                  1H
CPDPRG[2         waltz16
PCPD2              70.00 usec
PLW2         19.99799919 W
PLW12         0.49383000 W
PLW13         0.24839000 W

F2 - Processing parameters
SI                 32768
SF           150.9028085 MHz
WDW                   EM
SSB      0
LB                  1.00 Hz
GB       0
PC                  1.40
```

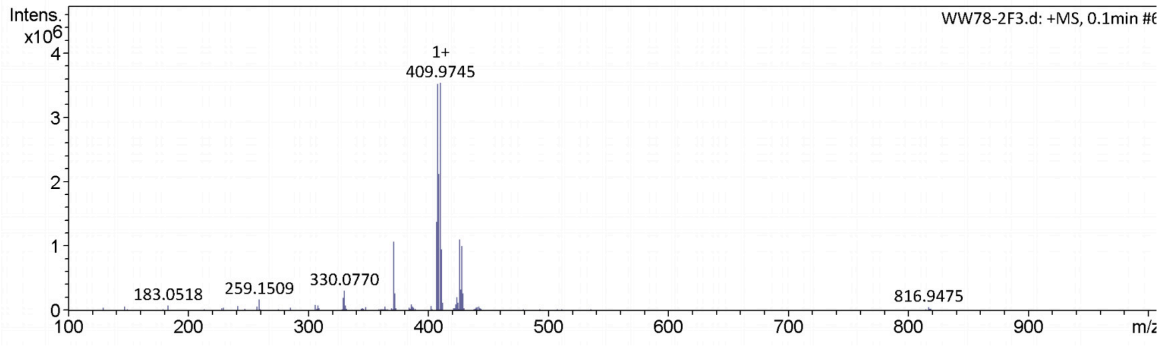

Compound TPIAnCN

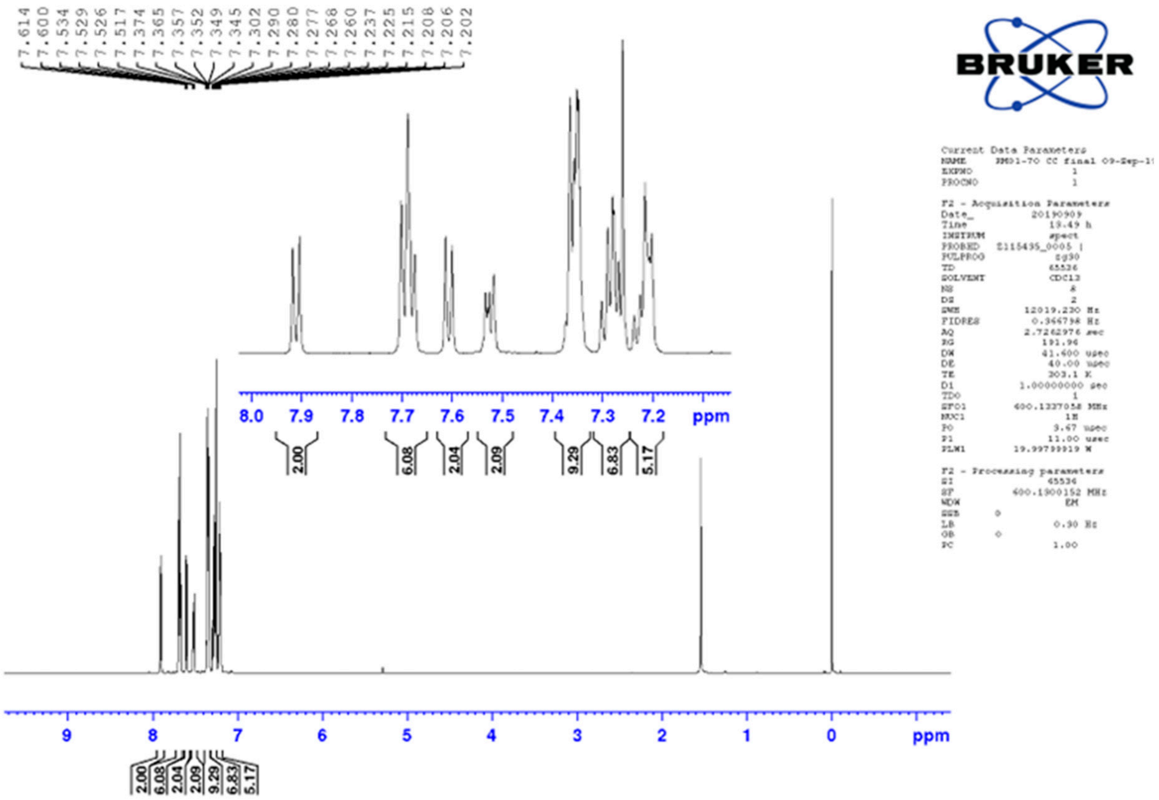

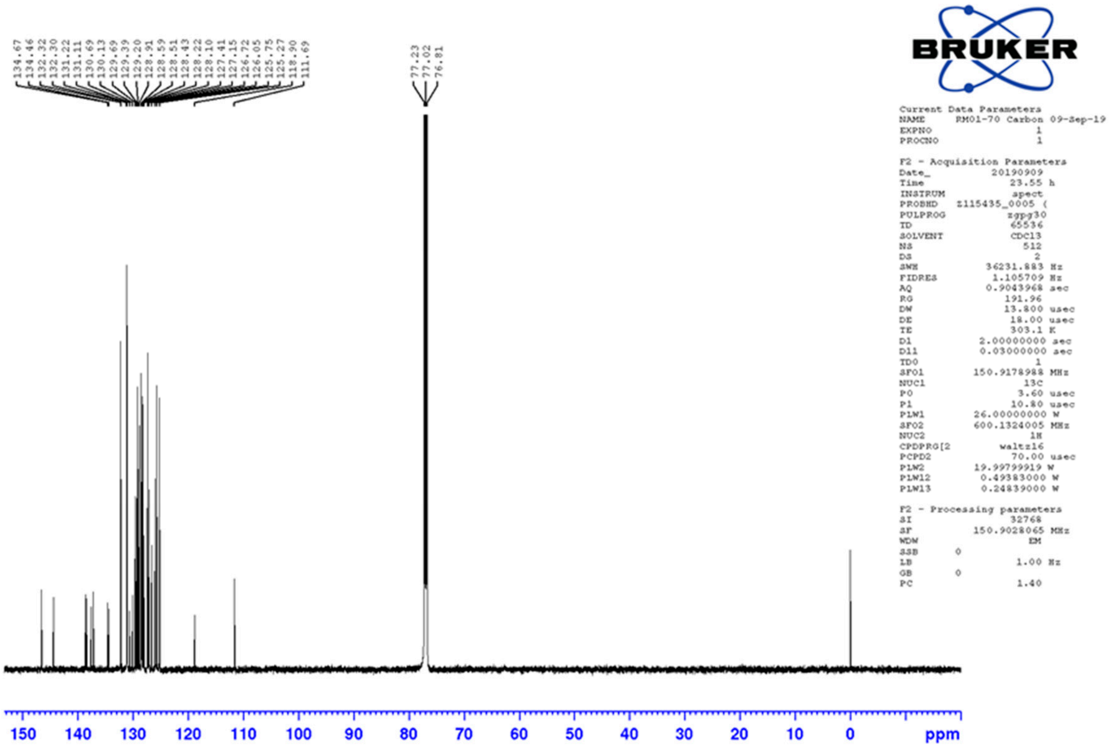

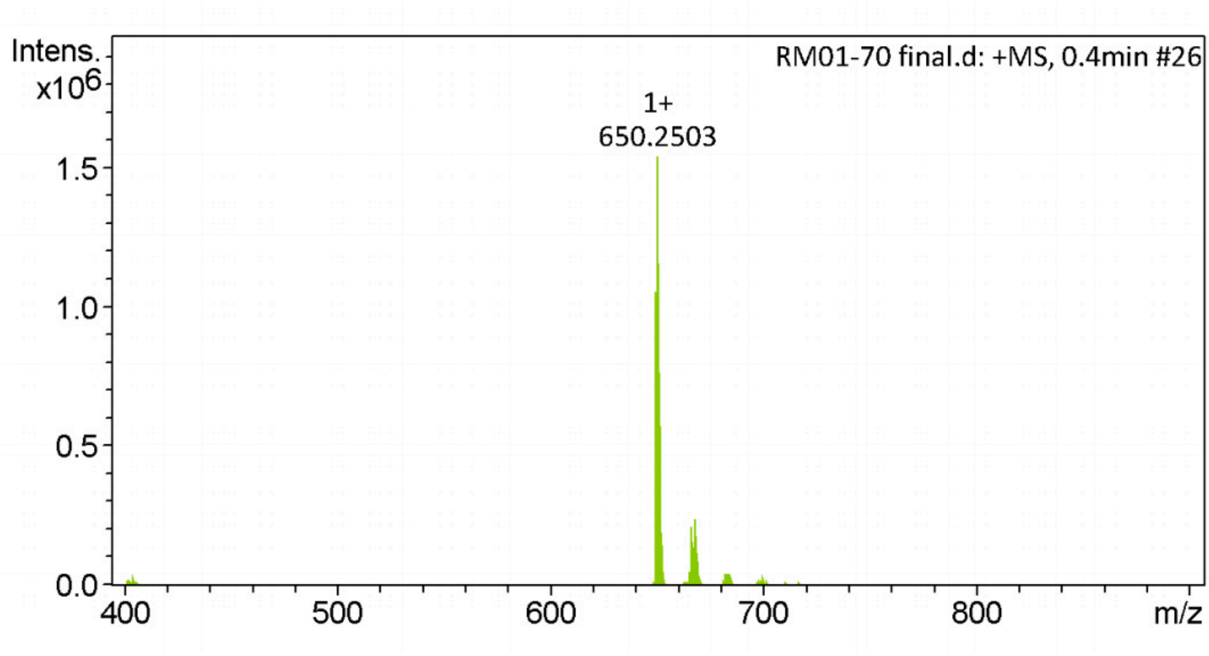

Compound TPIChCN

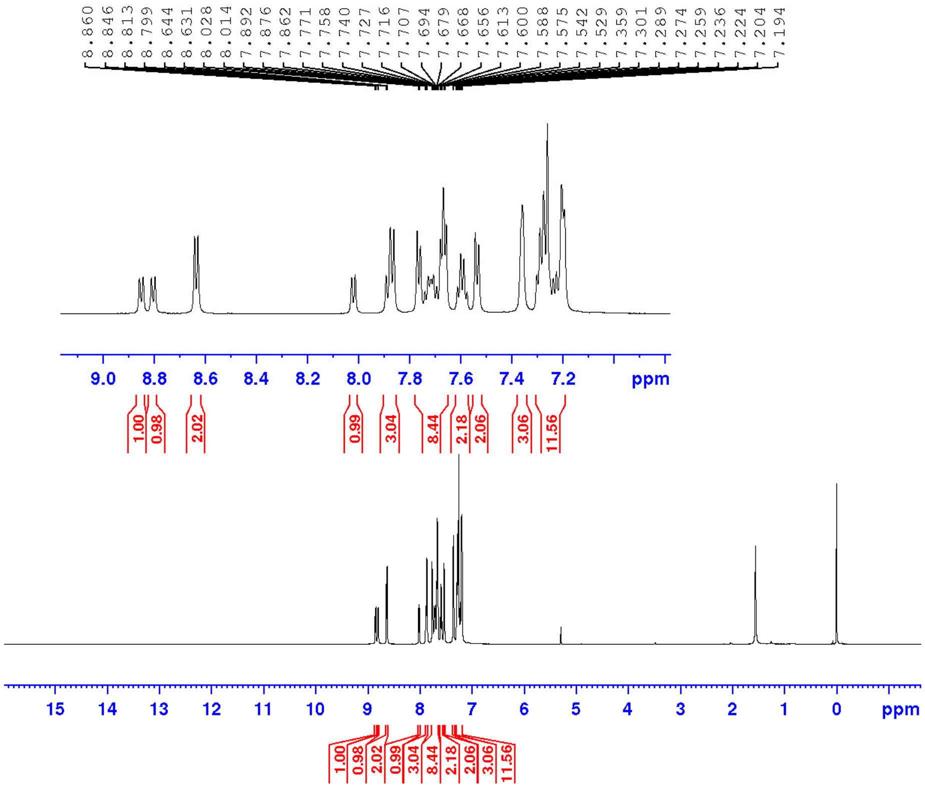


```
Current Data Parameters
NAME          WW84-1F2C2
EXPNO                  1
PROCNO                 1

F2 - Acquisition Parameters
Date_           20191101
Time               17.18 h
INSTRUM            spect
PROBHD   Z114607_0208 (
PULPROG             zg30
TD                 64902
SOLVENT            CDCl3
NS                     8
DS                     2
SWH            12019.230 Hz
FIDRES          0.370381 Hz
AQ             2.6999233 sec
RG                107.65
DW                41.600 usec
DE                  6.50 usec
TE                 303.1 K
D1            2.00000000 sec
TD0                    1
SFO1         600.1336008 MHz
NUC1                  1H
P0                  3.23 usec
P1                  9.70 usec
PLW1         23.00000000 W

F2 - Processing parameters
SI                 65536
SF           600.1300163 MHz
WDW                   EM
SSB      0
LB                  1.00 Hz
GB       0
PC                  1.00
```

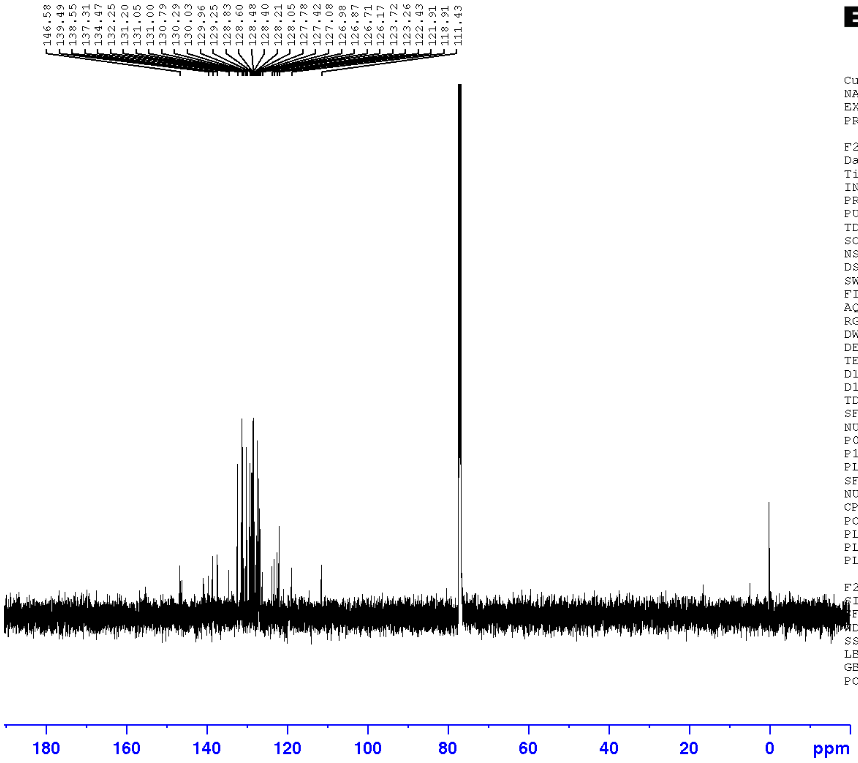

BRUKER

```
Current Data Parameters
NAME              WW84-1C1
EXPNO                    1
PROCNO                   1

F2 - Acquisition Parameters
Date_             20191102
Time                  3.07 h
INSTRUM              spect
PROBHD     Z114607_0208 (
PULPROG            zgpg30
TD                   65536
SOLVENT              CDCl3
NS                    2048
DS                       2
SWH              36231.883 Hz
FIDRES            1.105709 Hz
AQ               0.9043968 sec
RG                  191.96
DW                  13.800 usec
DE                    6.50 usec
TE                   303.2 K
D1              2.00000000 sec
D11             0.03000000 sec
TD0                      1
SFO1           150.9178988 MHz
NUC1                   13C
P0                    3.27 usec
P1                    9.80 usec
PLW1          100.69000244 W
SFO2           600.1324005 MHz
NUC2                    1H
CPDPRG[2           waltz16
PCPD2                70.00 usec
PLW2           23.00000000 W
PLW12           0.44165000 W
PLW13           0.22215000 W

F2 - Processing parameters
SI                   32768
SF             150.9028085 MHz
WDW                     EM
SSB        0
LB                    1.00 Hz
GB         0
PC                    1.40
```

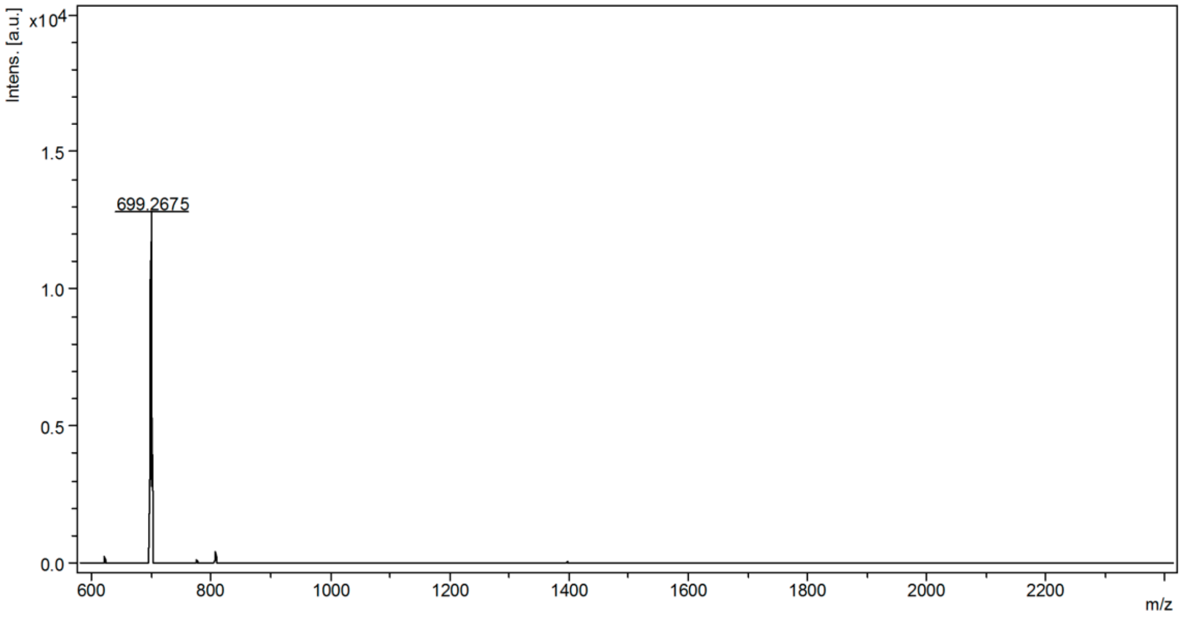

m/z	S/N	Quality Fac.	Res.	Intens.	Area
699.2675				2544	

Table S1 Crystallographic data of **TPIAnCN** and **TPIChCN**.

**Chemical structure**	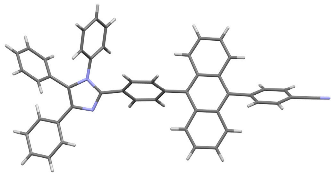	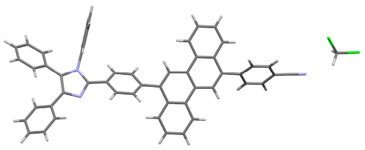
**CCDC Deposit Number**	**2048655**	**2011495**
Empirical formula	$C_{48}H_{31}N_3$	$C_{53}H_{35}Cl_2N_3$
Formula weight	649.76	784.74
Temperature/K	100.0	110.0
Crystal system	monoclinic	triclinic
Space group	$P2_1/c$	P-1
a/Å<br>b/Å<br>c/Å	6.0526(4)<br>14.6484(9)<br>38.294(3)	10.0312(6)<br>10.7769(7)<br>18.8593(11)
α/°<br>β/°<br>γ/°	90<br>93.888(3)<br>90	90.123(2)<br>101.607(2)<br>107.107(2)
Volume/Å^3	3387.4(4)	1904.6(2)
Z	4	2
ρ_{calc}g/cm^3	1.274	1.368
μ/mm^{-1}	0.573	0.215
F(000)	1360.0	816.0
Crystal size/mm^3	0.25 × 0.12 × 0.03	0.554 × 0.284 × 0.058
Radiation	CuKα (λ = 1.54178)	MoKα (λ = 0.71073)
2Θ range for data collection/°	4.626 to 99.484	3.962 to 52.744
Index ranges	$-5 \leq h \leq 5$,<br>$-13 \leq k \leq 14$,<br>$-37 \leq l \leq 37$	$-12 \leq h \leq 12$,<br>$-13 \leq k \leq 13$,<br>$-23 \leq l \leq 23$
Reflections collected	35796	74150
Independent reflections	3420 [R_{int} = 0.1802,<br>R_{sigma} = 0.1039]	7800 [R_{int} = 0.0510,<br>R_{sigma} = 0.0259]
Data/restraints/parameters	3420/0/460	7800/0/524
Goodness-of-fit on F^2	1.046	1.052
Final R indexes [I>=2σ (I)]	R_1 = 0.0634, wR_2 = 0.1071	R_1 = 0.0630, wR_2 = 0.1710
Final R indexes [all data]	R_1 = 0.1255, wR_2 = 0.1253	R_1 = 0.0749, wR_2 = 0.1788
Largest diff. peak/hole / e Å^{-3}	0.21/-0.24	0.82/-1.34

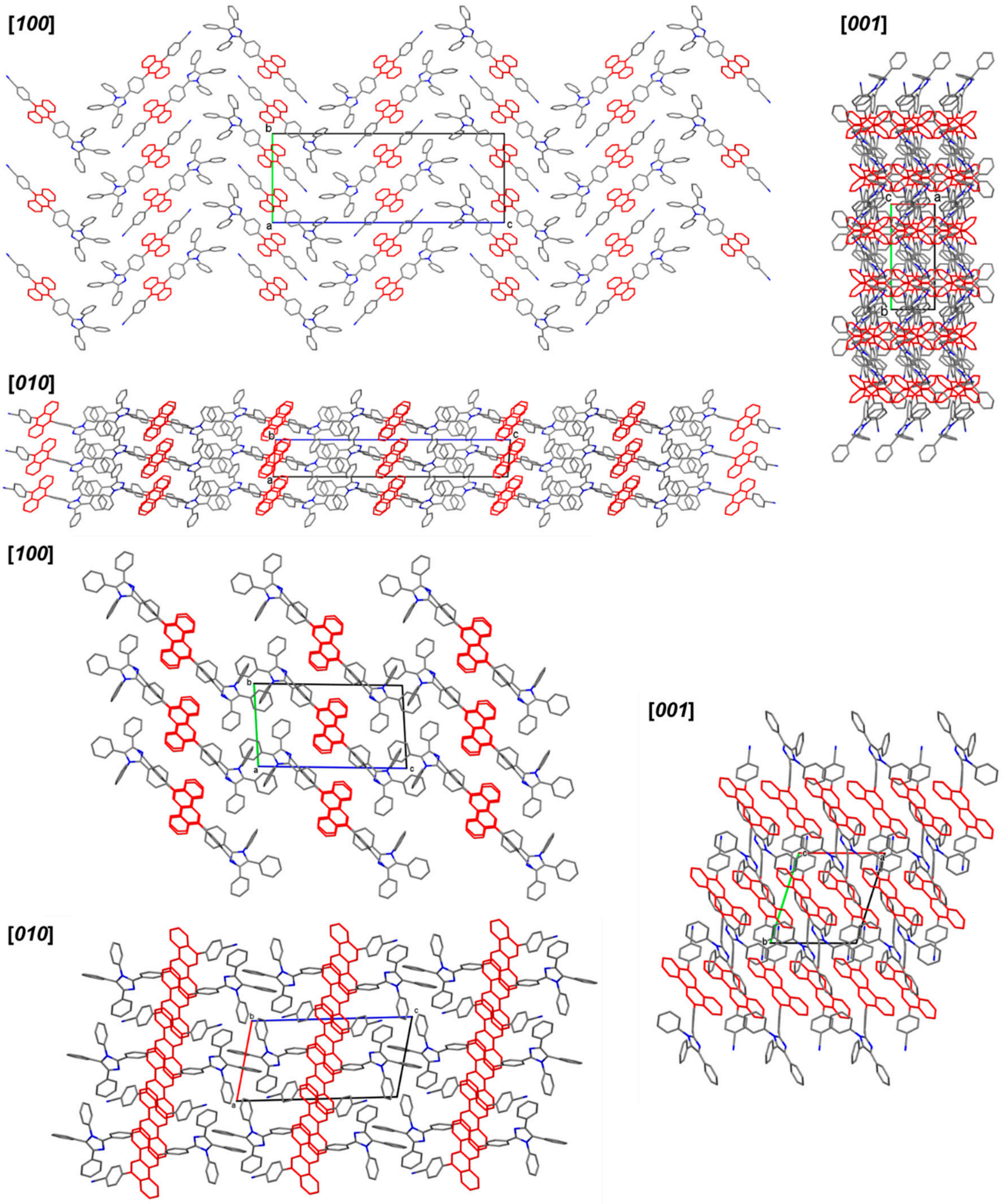

Figure S2. Crystal packing of (top) **TPIAnCN** and (bottom) **TPIChCN** along [*100*], [*010*] and [*001*] directions with red-highlighted structures of anthracene and chrysene cores, respectively. All hydrogen atoms and solvent residues were omitted for clarity.

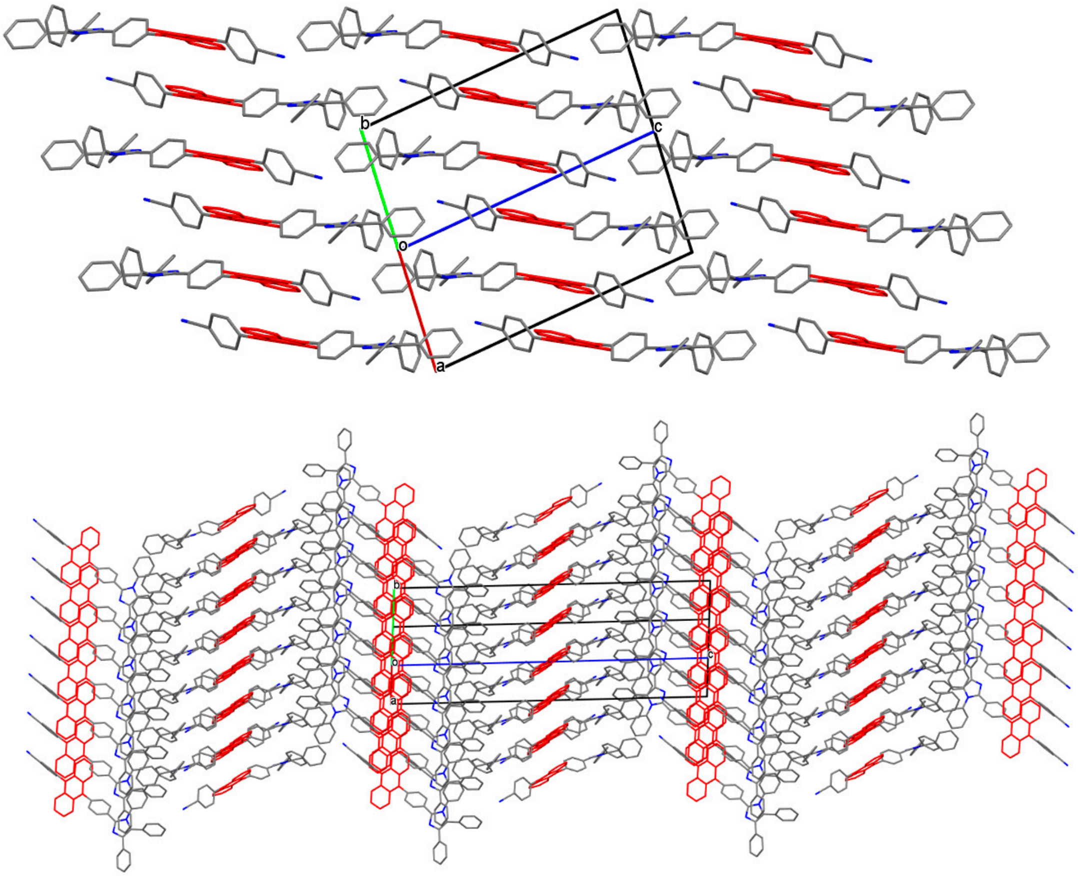

Figure S3. Packing structure of (top) **TPIAnCN** and (bottom) **TPIChCN** along [*110*] and [*210*] directions, respectively, with red-highlighted structures of anthracene and chrysene moieties. All hydrogen atoms and solvent residues were omitted for clarity.

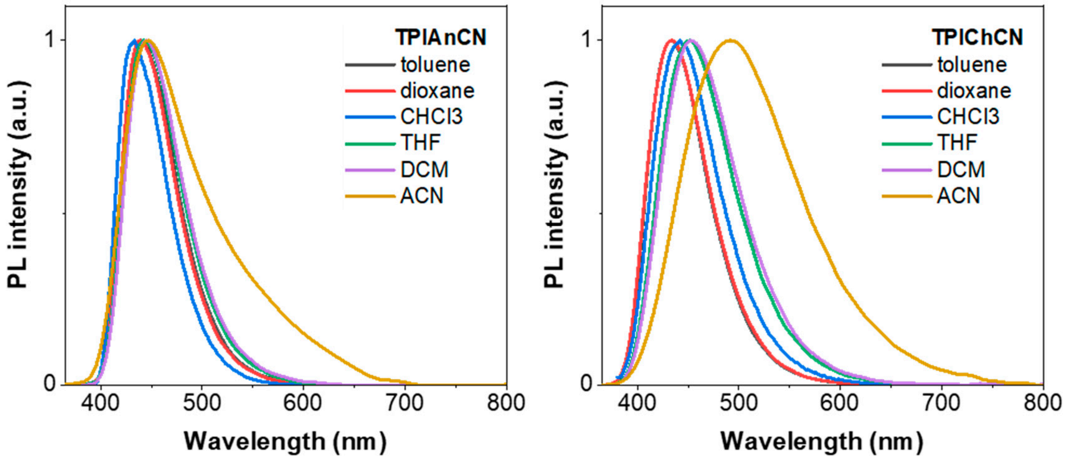

Figure S4. PL spectra in different solvents.

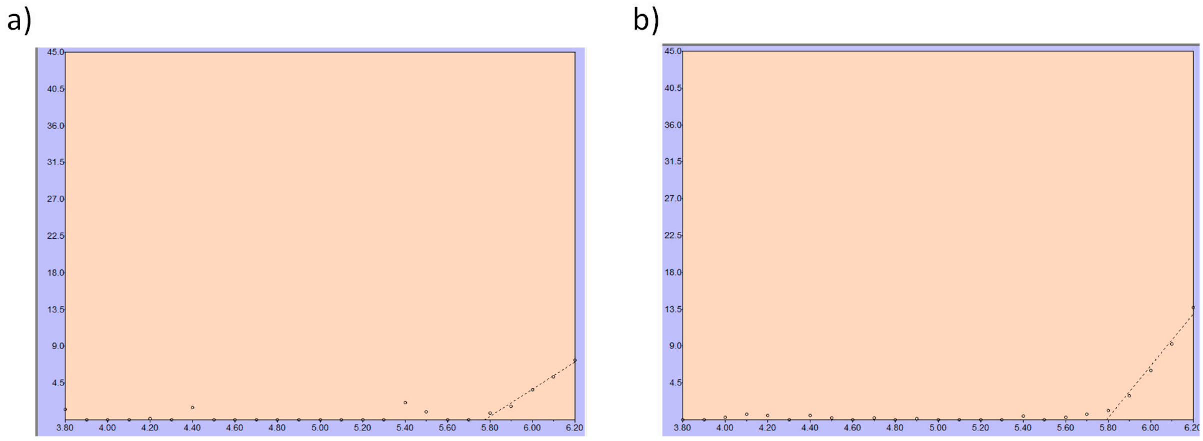

Figure S5. AC-2 plots of a) **TPIAnCN** and b) **TPIChCN**.

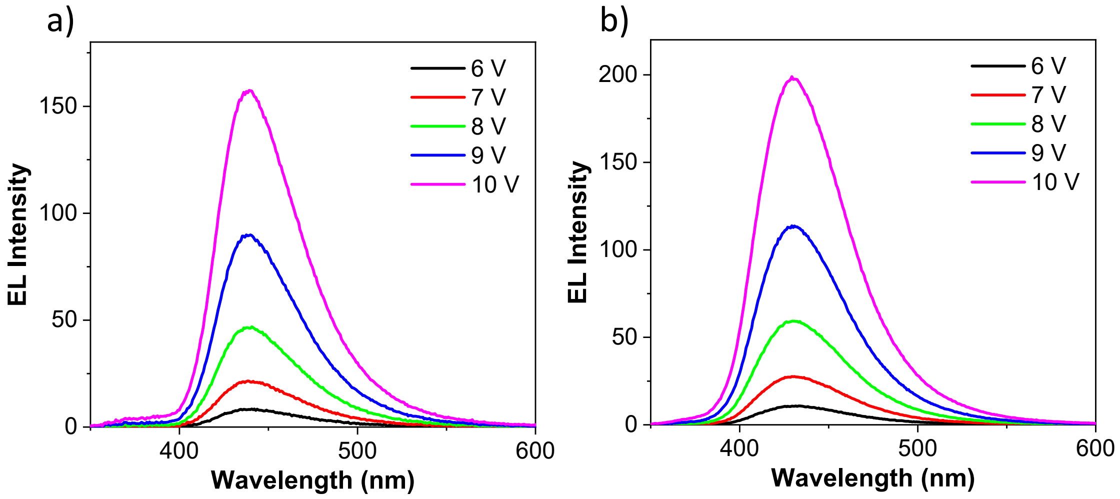

Figure S6. EL spectra under different applied voltages.
